# Tree-Based Machine Learning Model for Fluorescence Lifetime Prediction in Organic Compounds

**DOI:** 10.1007/s10895-026-04839-9

**Published:** 2026-06-19

**Authors:** Dragoș-Cătălin Vovea, Vasile Chiș

**Affiliations:** Faculty of Physics, Babeș-Bolyai University, Str. M. Kogălniceanu 1, Cluj-Napoca, RO- 400084 Romania

**Keywords:** Fluorescence lifetime prediction, Tree-based machine learning, Chromophores, Molecular descriptors, LightGBM, SHAP analysis

## Abstract

Fluorescence lifetime is a key photophysical property that reflects the excited-state decay dynamics of organic chromophores and is essential for applications in fluorescence imaging, sensing, optoelectronics, and materials screening. In this study, a tree-based machine-learning framework was developed to predict fluorescence lifetimes using chromophore–solvent data derived from the Deep4Chem database. Molecular structures were encoded through RDKit descriptors, Morgan and MACCS fingerprints, solvent descriptors, engineered interaction features, and photophysical variables, enabling structural, electronic, geometrical, and environmental effects to be captured in a unified descriptor space. Several regression algorithms were benchmarked, and LightGBM was selected as the final model owing to its balance of predictive accuracy, computational efficiency, scalability, and interpretability. The optimized model was evaluated using cluster-stratified 10-fold cross-validation, achieving a mean MAE of 0.8324 ± 0.0617 ns and a mean R² of 0.7523 ± 0.0317. Error analysis showed that the model performs most reliably in the intermediate lifetime range, where data density is highest, while larger deviations occur for sub-nanosecond and long-lifetime chromophores due to relative-error amplification, experimental uncertainty, and limited sample representation. External validation against TD-DFT benchmarks and independent experimental data further demonstrated that the model captures global fluorescence lifetime trends and can serve as a rapid screening tool rather than a replacement for detailed spectroscopy or quantum-chemical calculations. SHAP analysis revealed that fluorescence lifetime is mainly governed by quantum yield, molecular geometry, emission-related descriptors, topological indices, and fragment-level structural motifs, indicating that the model learns physically meaningful relationships between molecular structure, solvent environment, excited-state stabilization, and non-radiative relaxation.

**Supplementary Information:**

The online version contains supplementary material available at 10.1007/s10895-026-04839-9.

## Introduction

Fluorescence lifetime is a fundamental photophysical property that describes the average time a fluorophore remains in an excited state before returning to the ground state through photon emission [1, 2]. Because it is directly related to the balance between radiative and non-radiative decay processes, fluorescence lifetime is widely used in optoelectronic applications such as organic light-emitting diodes (OLEDs) and organic photovoltaics (OPVs), chemical and biological sensing, and biomedical imaging techniques such as fluorescence lifetime imaging microscopy (FLIM) [3–6]. However, experimental lifetime measurements often require specialized instrumentation, careful sample preparation, and relatively long acquisition times, which can limit the rapid screening and discovery of new chromophores [5, 6]. In addition, the synthesis and structural optimization of new fluorophores represent a major bottleneck in experimental screening workflows, since probe development often requires iterative molecular design, chemical modification, purification, and photophysical characterization before suitable candidates can be identified [7].

To address these limitations, predictive computational approaches, particularly machine learning (ML), have emerged as efficient tools for photophysical property estimation and high-throughput molecular screening [8–11] In this work, we develop a tree-based ML model to predict fluorescence lifetimes for chemically diverse chromophores using the Deep4Chem dataset [8]. The model combines molecular descriptors derived from chromophore and solvent structures with experimental photophysical information, enabling rapid lifetime prediction without the need for exhaustive experimental measurements. In addition to prediction, the framework supports interpretation through feature-importance and SHAP-based analyses [12], allowing the structural and electronic factors associated with fluorescence lifetime modulation to be identified.

By providing fast and interpretable lifetime estimates, the proposed approach can support experimental workflows by prioritizing promising fluorophores for synthesis, measurement, or further quantum-chemical investigation. In this sense, the ML model does not replace experimental spectroscopy or TD-DFT calculations [13], but acts as a low-cost screening layer that can guide more detailed photophysical analysis. Such integration of data-driven prediction with experimental and theoretical methods may accelerate the discovery and optimization of fluorescent materials for imaging, sensing, OLEDs, OPVs, and related photonic applications.

Recent studies have demonstrated the potential of machine learning for predicting fluorescence-related properties, including emission wavelengths, quantum yields, and excitation-dependent observables [9–11]. In particular, Souza et al. [10] used the Deep4Chem [8] database to predict fluorescence emission maxima and quantum yields by combining structure-based molecular descriptors with solvent information. Their results showed that molecular and environmental descriptors can reproduce key photophysical observables, supporting the use of Deep4Chem as a valuable benchmark for data-driven fluorescence modeling [8, 10].

However, most previous ML studies have focused mainly on steady-state optical properties, whereas fluorescence lifetime has received comparatively less attention. Lifetime is physically distinct from emission wavelength or quantum yield because it reflects the kinetic competition between radiative and non-radiative excited-state decay pathways. As a result, it is strongly connected to excited-state dynamics, molecular rigidity, solvent relaxation, vibronic coupling, and internal conversion processes [1, 2].

The present work therefore extends previous Deep4Chem-based ML studies by specifically targeting fluorescence lifetime prediction. Whereas emission wavelength prediction primarily captures electronic transition energies, fluorescence lifetime prediction offers complementary information on excited-state relaxation dynamics. By integrating chromophore descriptors, solvent-dependent features, and semiempirical quantum-chemical descriptors, the model aims not only to improve predictive performance but also to identify the molecular factors most strongly associated with lifetime modulation.

From a computational perspective, fluorescence lifetime prediction is also more challenging than the prediction of excitation or emission energies. Time-dependent density functional theory (TD-DFT) is widely used to model excited-state properties because it provides a physics-based description of electronic excitations and radiative transitions [13]. However, TD-DFT calculations remain computationally demanding for large-scale screening and can show reduced accuracy for charge-transfer states, strong solvent relaxation, conical intersections, and non-radiative decay pathways. Since fluorescence lifetime depends on both radiative and non-radiative relaxation mechanisms, ML models trained on experimental lifetimes offer a complementary route for rapidly capturing complex structure–property trends that are difficult to model efficiently using electronic-structure methods alone [9–11].

Machine learning approaches offer a complementary strategy for addressing these limitations. Once trained, ML models can evaluate large molecular libraries at a fraction of the computational cost required for quantum-chemical calculations, making them especially attractive for high-throughput fluorophore discovery. Recent studies have further demonstrated that ML methods can implicitly learn complex structure–property relationships associated with excited-state relaxation, solvent effects, and photophysical behavior when sufficiently diverse training datasets are available [10]. Nevertheless, ML models also possess important limitations, including reduced interpretability, sensitivity to dataset quality, and limited extrapolation capability outside the chemical domain represented in the training data.

Consequently, fluorescence lifetime prediction represents an important intermediate problem situated between conventional steady-state spectral prediction and fully dynamical excited-state simulations. In this context, machine learning frameworks trained on experimentally measured lifetimes may provide an effective route toward capturing complex photophysical trends that remain computationally expensive or insufficiently described by TD-DFT-based methodologies.

In this context, the present work extends previous Deep4Chem-based machine learning studies [10] by specifically targeting fluorescence lifetime prediction. This analysis helps fill an important gap in computational assessments of fluorophore performance by linking experimentally accessible lifetime measurements to a broader set of molecular and photophysical descriptors. Furthermore, by integrating structural descriptors, solvent-dependent features, and semiempirical quantum-chemical descriptors, this study seeks not only to improve predictive performance but also to identify the molecular characteristics most strongly associated with lifetime modulation. Such insights are particularly relevant for the rational design of fluorophores for sensing, imaging, and optoelectronic applications, where fluorescence lifetime often serves as a critical functional parameter beyond conventional steady-state observables.

## Materials and Methods

### Model Selection

The choice of a tree-based ensemble model was motivated by the moderate size and tabular nature of the dataset. Previous benchmarking studies have shown that gradient boosting and other tree-based models often outperform deep neural networks on medium-sized tabular datasets, particularly when the number of samples is limited [14]. This makes LightGBM [15] appropriate for chemically complex descriptor spaces containing physicochemical descriptors, molecular fingerprints, solvent variables, and engineered interaction features.

To support model selection, an initial benchmarking step was performed using the LazyPredict framework, which allowed multiple regression algorithms to be compared under identical preprocessing conditions [16]. The results reported in Supplementary data, showed that nonlinear ensemble methods clearly outperformed linear models. Classical regressors such as Ridge, Lasso, and ElasticNet showed lower predictive capability, whereas ensemble-based models achieved substantially higher R² values, confirming the nonlinear character of fluorescence lifetime prediction.

Among the tested models, ExtraTreesRegressor produced the highest benchmark accuracy, followed by HistGradientBoostingRegressor, LightGBM, and RandomForestRegressor. Although ExtraTrees yielded a slightly higher R² in this initial screening, LightGBM was selected for the final workflow because it offered the best compromise between accuracy, computational efficiency, scalability, and interpretability. Overall, the benchmarking results indicate that fluorescence lifetime depends on nonlinear interactions between molecular structure, solvent properties, and photophysical descriptors, which are more effectively captured by ensemble tree-based methods than by conventional linear models [17].

### Data and Descriptors Generation

The dataset employed in this work was obtained from the publicly available Deep4Chem repository, a curated collection of photophysical and chemical data for organic chromophores compiled from experimental literature sources [8]. Deep4Chem provides standardized chromophore, solvent, and spectroscopic information, making it suitable for data-driven photophysical modeling and machine learning applications [8].

Each entry contains the chromophore structure and the corresponding solvent environment encoded as SMILES string [8, 18]. This dual representation enables both solute and solvent information to be included in the descriptor space, which is important because solvent polarity, hydrogen bonding, dielectric relaxation, and excited-state stabilization can strongly influence fluorescence lifetime, quantum yield, and non-radiative decay pathways [1, 2].

In addition to structural information, the dataset reports experimentally measured photophysical properties, including absorption and emission maxima, fluorescence lifetime, fluorescence quantum yield, absorption and emission full width at half maximum, and the logarithm of the molar extinction coefficient [8]. These quantities provide information on electronic transition energies, radiative efficiency, spectral broadening, and excited-state relaxation behavior.

Deep4Chem covers a chemically diverse range of fluorophores, including aromatic chromophores, donor–acceptor systems, heterocycles, and extended conjugated molecules measured across solvents with different polarity and hydrogen-bonding properties. The complete database contains 20,236 measurements corresponding to approximately 7,016 unique chromophores in 365 solvents. This combination of molecular structure, solvent identity, and experimental photophysical data makes the dataset well suited for modeling structure–property relationships associated with fluorescence lifetime and solvent-dependent photophysical behavior.

Because the database is assembled from multiple literature sources, variability can arise from differences in experimental conditions, instrumental setups, sample preparation, and reporting standards. Therefore, data cleaning, normalization, feature engineering, and feature selection were applied before model development to improve consistency and reduce the effect of incomplete or heterogeneous entries [17, 19]. A summary of the initial dataset features is presented in Table 1.

**Table 1:** Deep4Chem initial columns

Column Name	Unit	Data Type	Description
Tag	—	Float	Index or numbering of the data points
Chromophore	—	String	SMILES representation of the chromophore structure
Solvent	—	String	SMILES representation of the solvent structure
Absorption max	nm	Float	Maximum absorption wavelength
Emission max	nm	Float	Maximum emission wavelength
Lifetime	ns	Float	Fluorescence lifetime
Quantum yield	—	Float	Photoluminescence quantum yield
log(ε / mol⁻¹ dm³ cm⁻¹)	—	Float	Logarithm of the molar extinction coefficient
abs FWHM	cm⁻¹	Float	Absorption bandwidth (full width at half maximum)
emi FWHM	cm⁻¹	Float	Emission bandwidth (full width at half maximum)
abs FWHM	nm	Float	Absorption bandwidth (full width at half maximum)
emi FWHM	nm	Float	Emission bandwidth (full width at half maximum)
Molecular weight	g mol⁻¹	Float	Molecular weight of the chromophore
Reference	—	String	DOI of the source document

The original Deep4Chem dataset contained approximately 20,236 entries [8]. For the purpose of this study, we focused exclusively on entries with measured fluorescence lifetime values, as this property is the primary target of our modeling efforts. This filtering reduced the dataset to approximately 6500 entries. After additional cleaning, descriptor generation, and consistency checks, the final curated dataset contained approximately 5000 complete chromophore–solvent entries suitable for model training. These pre-selection steps were necessary to reduce the influence of missing values, incomplete measurements, and inconsistencies arising from literature-derived experimental data.

Chromophore and solvent SMILES strings were converted into numerical molecular representations using the RDKit cheminformatics framework [20]. The computed descriptors covered several complementary categories. Constitutional descriptors described molecular composition, including atom counts, heteroatom content, bond counts, and ring statistics. Topological descriptors encoded molecular connectivity and shape using indices such as Kappa shape descriptors, Balaban-type connectivity measures, and related graph-based properties [20, 21]. Electronic descriptors captured polarity, lipophilicity, charge distribution, hydrogen-bonding capacity, and polar surface area, while three-dimensional descriptors described molecular volume, accessible surface area, radius of gyration, bond geometry, and dihedral-angle distributions derived from RDKit-generated conformations [20, 22].

The calculated descriptors provide a chemically meaningful description of both the chromophore and its environment. Correlation and feature-importance analyses indicated that quantum yield, molecular geometry, polarity-related descriptors, and conjugation-sensitive features are among the variables most strongly associated with fluorescence lifetime, consistent with classical fluorescence theory [1, 2]. Although chromophore-intrinsic descriptors remained dominant, solvent descriptors also contributed measurably to model predictions, supporting the need to encode solvent structure explicitly. This combined representation allows the model to account not only for intrinsic molecular structure, but also for environmental effects that modulate excited-state stabilization and relaxation kinetics.

To further enrich the descriptor space, predicted pKa values were calculated for each chromophore using a separate machine-learning model based on RDKit descriptors [23]. This descriptor was included because protonation equilibria and charge-state distributions can influence fluorescence behavior, particularly in chromophores containing ionizable functional groups. Variations in protonation state may alter electronic structure, charge-transfer character, and solvent interactions, which can in turn affect excited-state relaxation pathways. Although pKa did not emerge as one of the most influential descriptors in the present dataset, it was retained as a chemically meaningful variable that may provide additional predictive value for pH-sensitive fluorophore families.

Molecular fingerprints were also incorporated to capture structural patterns not fully represented by conventional physicochemical descriptors. Morgan fingerprints were generated using a 512-bit representation, providing a compact encoding of local atomic environments and molecular topology [24]. In this approach, atom-centered neighborhoods are iteratively expanded and hashed into binary identifiers, so that each bit reflects the presence of a characteristic substructure. The 512-bit length was selected as a compromise between structural resolution, computational efficiency, and feature sparsity.

In parallel, MACCS fingerprints were included in their standard 167-bit form to provide complementary and chemically interpretable structural descriptors [20]. Unlike Morgan fingerprints, which are algorithmically generated, MACCS keys correspond to predefined substructural patterns such as functional groups, heteroatom environments, aromatic motifs, and ring systems. The combined use of Morgan and MACCS fingerprints therefore allows the model to capture both detailed local topology and interpretable fragment-level chemistry.

This fingerprint-based strategy follows previous fluorescence prediction studies, including the work of Souza et al., who showed that molecular fingerprints and solvent information can improve machine learning models for photophysical properties [10]. In the present study, this approach was extended by incorporating both chromophore and solvent fingerprints together with RDKit descriptors and engineered interaction features. The final descriptor space therefore combines physicochemical, topological, fragment-based, and solvent-dependent information into a high-dimensional but chemically meaningful representation for fluorescence lifetime prediction.

In addition to descriptor construction, unsupervised clustering was used to explore the chemical space and support structure-aware data analysis. K-means clustering [25, 26] was applied to the full descriptor representation, including RDKit descriptors and fingerprint-based features, to group molecules according to structural and physicochemical similarity. This analysis provided a global view of dataset diversity and helped identify chemically meaningful regions of the descriptor space. The resulting cluster assignments were subsequently used to support interpretation and stratified model evaluation, ensuring that different structural families were more evenly represented across validation folds.

To quantify clustering quality, the Within-Cluster Sum of Squares (WCSS), also referred to as inertia, was calculated [25]. WCSS measures the compactness of the clustering solution by summing the squared Euclidean distances between each data point and the centroid of the cluster to which it belongs. Lower WCSS values indicate more compact clusters; however, increasing the number of clusters will naturally reduce WCSS. Therefore, the elbow method was used to identify a suitable compromise between cluster compactness and model simplicity [26].

The inertia is defined as:

$$\:Inertia={\sum\:}_{k=1}^{K}{\sum\:}_{{x}_{i}\in\:{C}_{k}}^{\:}\parallel\:{x}_{i}-{\mu\:}_{k}{\parallel\:}^{2}\:\:\:\:\:\:\:\:\:\:\:\:\:\:\:\:\:\:\:\:\:\:\:\:\:\:\:\:\:\:\:\:\:\:\:\:\:\:\:\:\:\:\:\:\left(1\right)$$

where *K* is the number of clusters, *C*_*k*_ is the set of data points assigned to cluster K, *x*_*i*_ is the feature vector of molecule *i*, µ_k_ is the centroid of cluster *k.*

**Fig. 1:**
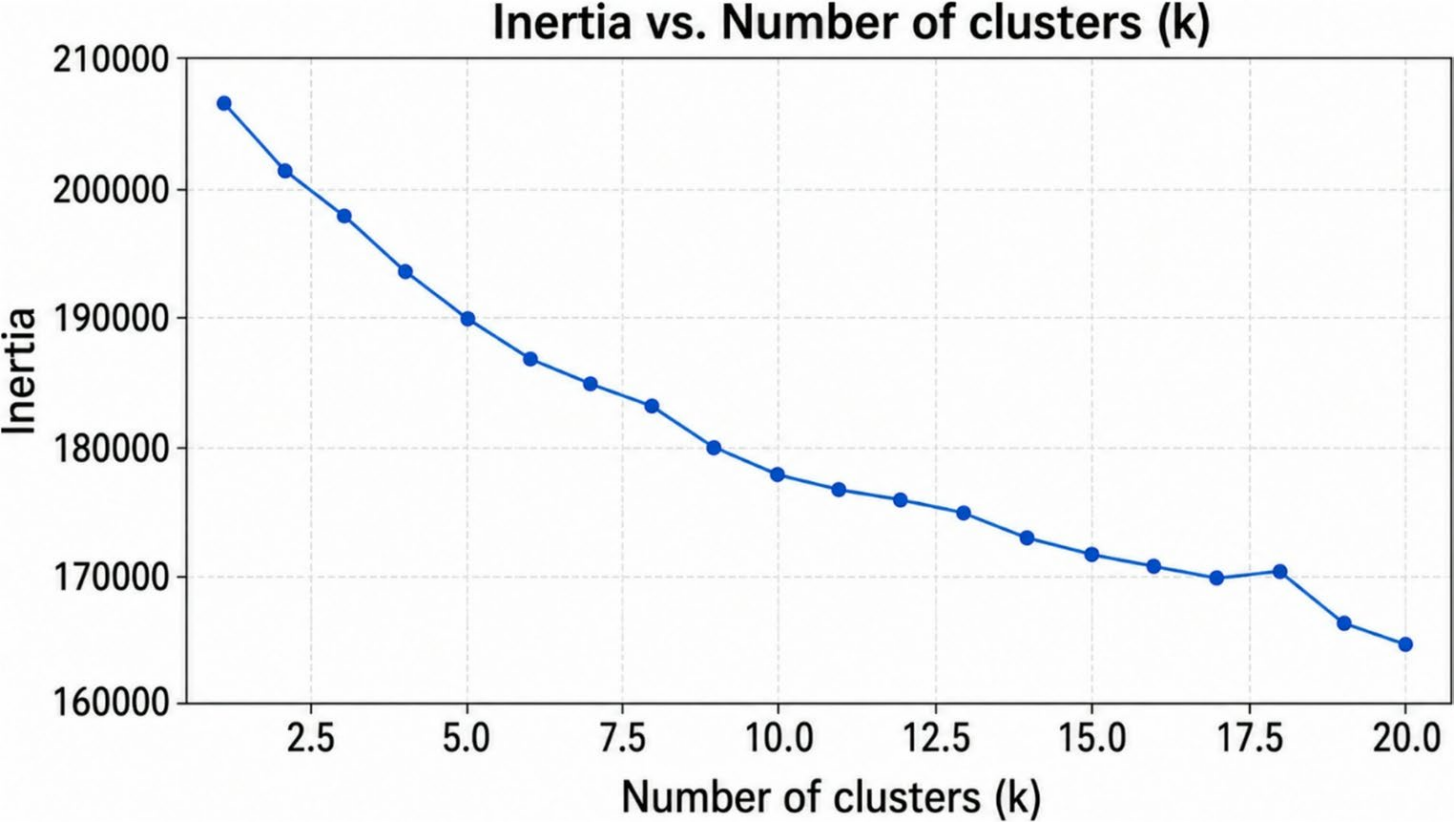
Elbow method applied for identifying molecules categories via unsupervised algorithm Kmeans

As shown in Fig. 1, several possible elbow points can be observed. Among them, k = 13 provided the best compromise between cluster separation and model stability. After selecting the number of clusters, each molecule was assigned to a cluster label representing its structural and physicochemical neighborhood in descriptor space. This cluster label was then used for stratified cross-validation to ensure that each fold contained balanced proportions of the identified molecular groups, thereby reducing the effect of structural imbalance on model performance.

The analysis showed that the chemical space is dominated by a limited set of recurring structural motifs that vary systematically across the identified clusters. Rather than being defined by many unrelated fragments, each cluster is characterized by combinations of aromatic ring systems, heteroatom-rich environments, polar functional groups, and solvent-accessible motifs. These recurring patterns act as structural signatures associated with fluorescence lifetime behavior.

Clusters associated with longer fluorescence lifetimes were generally enriched in rigid aromatic and π-conjugated ring systems, suggesting enhanced excited-state stabilization and reduced vibrational relaxation. In contrast, clusters dominated by heteroatom-rich, highly polar, or strongly functionalized motifs tended to show shorter lifetimes, consistent with stronger solvent coupling, internal conversion, and non-radiative decay processes [1, 2]. Several clusters also contained halogenated or strongly substituted aromatic motifs, which may indicate the contribution of heavy-atom effects and enhanced intersystem crossing pathways.

The clustering analysis further indicates that fluorescence lifetime is not governed by isolated molecular fragments alone, but by the combined effects of aromaticity, rigidity, heteroatom content, polarity, and solvent interactions. Aromatic and fused-ring motifs generally support longer-lived excited states through increased conjugation and structural rigidity, whereas flexible topologies and electron-rich polar environments favor faster excited-state deactivation.

**Fig. 2:**
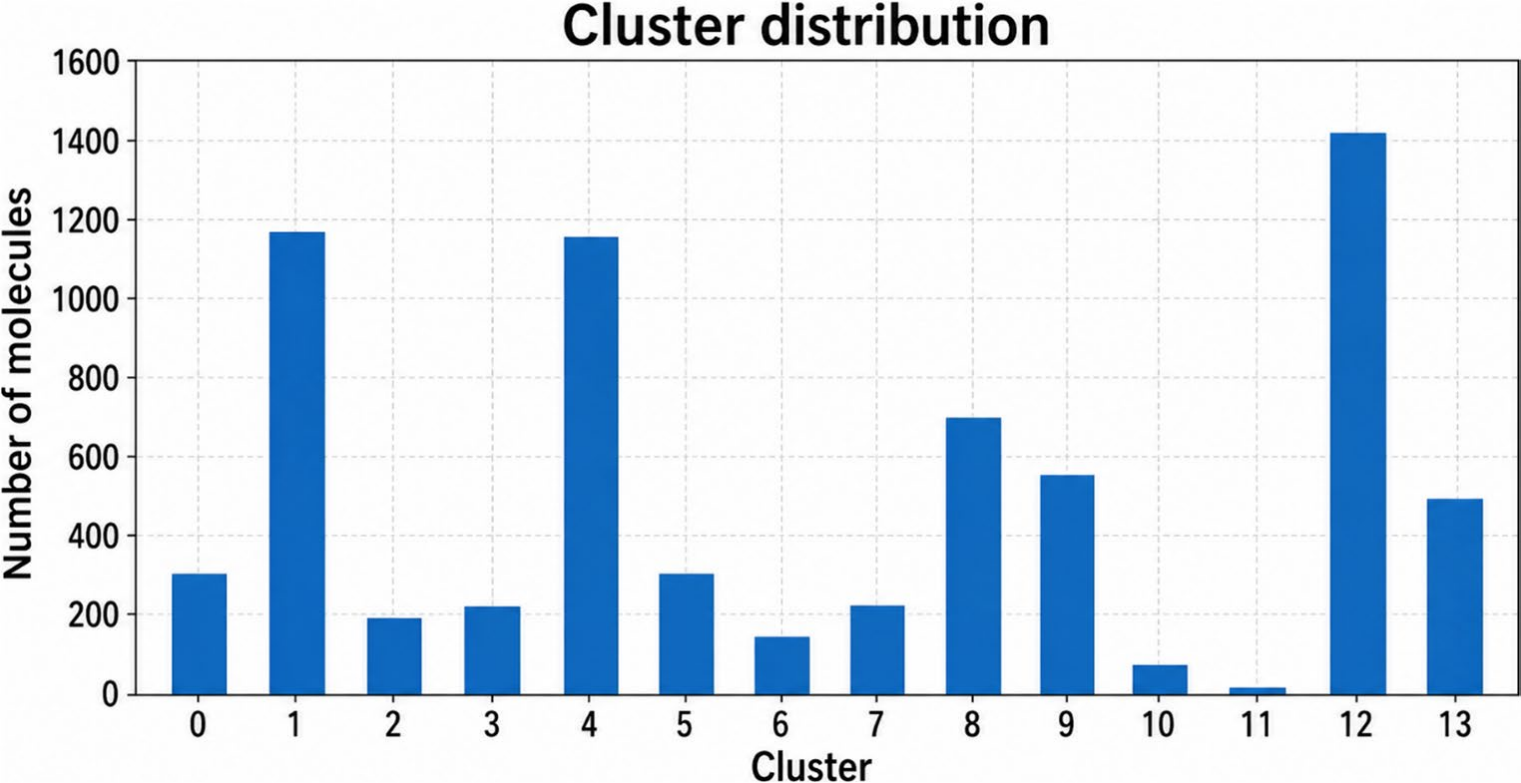
Cluster distribution among initial database

The cluster population distribution shown in Fig. 2 supports this interpretation. The largest clusters, particularly Clusters 12, 1, and 4, correspond mainly to aromatic and moderately conjugated molecular families, indicating that these structural types occupy the most populated regions of the fluorescence-lifetime chemical space. In contrast, smaller clusters such as 10 and 11 represent more specialized chemotypes enriched in heteroatom-rich, sulfur-containing, or strongly polar motifs. These clusters occupy sparser regions of the descriptor manifold and are generally associated with shorter fluorescence lifetimes.

Overall, cluster interpretation shows that a relatively small number of chemically meaningful structural patterns accounts for a substantial part of the photophysical variability in the dataset. These recurring motifs define the chemical identity of each cluster and provide an interpretable link between molecular structure and fluorescence lifetime behavior. The main cluster characteristics, dominant structural motifs, and associated photophysical trends are summarized in Table 2, together with the cluster distribution shown in Fig. 2.

**Table 2:** Identified clusters interpretations

Cluster	Physicochemical Class	Dominant MACCS motifs (interpreted)	Structural interpretation	Photophysical implication
0	Heavy, non-polar, rigid, long lifetime	aromatic rings (a), ring systems ([R]), conjugated scaffolds	highly rigid aromatic frameworks with strong π-conjugation and constrained geometry	stabilized excited states, reduced vibrational loss → long lifetime
1	Light, non-polar, flexible, long lifetime	aromatic rings, ring fragments, heteroatoms (N)	mixed aromatic systems with moderate flexibility and moderate heteroatom content	balanced radiative decay, moderately stable excited states
2	Light, polar, flexible, short lifetime	heteroatom–heteroatom bonds, S/O functional groups	polar, heteroatom-rich solvent-exposed structures	strong solvent coupling → non-radiative decay
3	Heavy, polar, rigid, short lifetime	aromatic rings + N/O networks	rigid heteroatom-rich chromophores	internal conversion dominates despite rigidity
4	Light, non-polar, rigid, short lifetime	aromatic rings, halogen motifs (Cl, F), ring scaffolds	rigid aromatic systems with halogen substitution	heavy-atom effects + non-radiative decay pathways
5	Heavy, polar, rigid, short lifetime	carbonyls, amides, heteroatom-rich scaffolds	strongly functionalized rigid chromophores	strong charge transfer + fast deactivation
6	Heavy, polar, rigid, short lifetime	aromatic + dense N/O networks	highly functionalized rigid macrostructures	high internal conversion efficiency
7	Heavy, polar, rigid, short lifetime	aromatic rings + nitrogen-containing fragments	extended conjugated rigid frameworks	structural rigidity insufficient to suppress quenching
8	Light, non-polar, flexible, long lifetime	aromatic rings + small heteroatoms (N)	flexible aromatic systems with moderate polarity	reduced non-radiative decay → longer lifetime
9	Heavy, non-polar, flexible, short lifetime	aromatic systems + heteroatom bonds	mixed polarity rigid-to-flexible systems	structural heterogeneity promotes relaxation
10	Light, polar, flexible, short lifetime	N/S functional groups, amines	heteroatom-rich polar fragments	strong n→π* relaxation → quenching
11	Light, non-polar, flexible, short lifetime	small heterocycles, heteroatom bonds	small flexible heteroatom-containing fragments	fast relaxation, weak emission stability
12	Light, non-polar, flexible, short lifetime	aromatic rings + heteroatoms	aromatic but solvent-sensitive structures	solvent-driven deactivation dominates
13	Light, non-polar, rigid, short lifetime	ring systems + oxygen linkers	rigid but electronically weakly stabilized scaffolds	structural rigidity without electronic stabilization

### Data Preprocessing and Exploration

Feature engineering [17] was applied to improve predictive performance and to better represent the nonlinear physicochemical relationships that govern fluorescence lifetime. In addition to the original molecular descriptors, transformed variables were generated using products, ratios, logarithmic transformations, powers, and squared terms. These engineered features were included because fluorescence behavior often depends on coupled effects between molecular size, polarity, topology, solvent environment, and electronic structure, rather than on isolated descriptors alone [1, 2].

Particular attention was given to chromophore–solvent interaction terms. Fluorescence lifetime is influenced not only by the intrinsic structure of the chromophore, but also by solvent-dependent effects such as dielectric stabilization, hydrogen bonding, solvent relaxation, and modulation of non-radiative decay pathways. Therefore, interaction descriptors combining chromophore and solvent properties, such as polarity–polarity, aromaticity–solvent, and rigidity–solvent terms, were introduced to help the model capture environment-dependent behavior.

Additional intramolecular descriptors were generated from ratios and products involving molecular weight, polar surface area, conjugation-related descriptors, and rigidity indices. These features were intended to approximate effects related to electronic delocalization, steric restriction, conformational flexibility, and charge-transfer character, all of which are relevant to excited-state relaxation dynamics [1, 2].

**Fig. 3:**
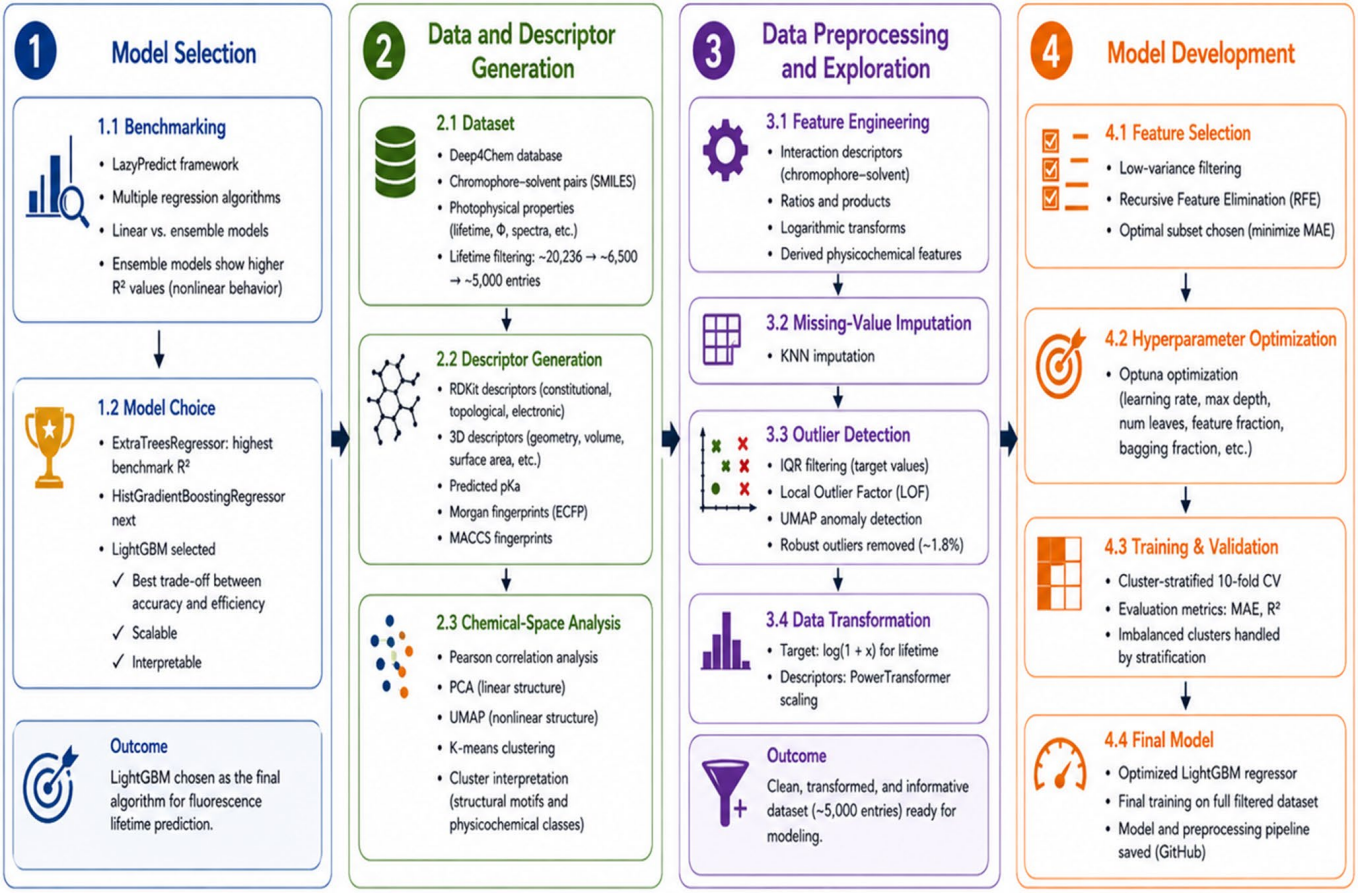
Overview of the machine-learning workflow developed for fluorescence lifetime prediction. The workflow consists of four stages: (1) Model Selection through benchmarking of multiple regression algorithms and selection of LightGBM; (2) Data and Descriptor Generation including dataset curation, descriptor and fingerprint calculation, predicted pKa estimation, and chemical-space analysis; (3) Data Preprocessing and Exploration comprising feature engineering, KNN imputation, outlier detection using IQR, LOF, and UMAP, and data transformation; and (4) Model Development through feature selection, Optuna hyperparameter optimization, cluster-stratified cross-validation, and construction of the final LightGBM fluorescence lifetime prediction model

Overall, feature engineering increased the expressiveness of the descriptor space and allowed the model to capture nonlinear dependencies that may not be represented by raw descriptors alone. The resulting feature set therefore combines data-driven transformations with chemically motivated photophysical information. The complete list of descriptors and engineered variables used in the final model is provided in the Supplementary data and a brief description of the whole process is provided in Fig. 3.

The data preprocessing pipeline included missing-value imputation, normalization, and outlier filtering. Missing descriptor values were imputed using a K-nearest neighbors (KNN) approach, in which missing entries are estimated from the most similar molecules in descriptor space [17].

To reduce the influence of extreme values during model training, a multi-stage filtering strategy was applied. This procedure combined target-based filtering of fluorescence lifetimes, statistical outlier detection, and structure-aware anomaly detection in the molecular descriptor space. First, the lifetime distribution was refined using the interquartile range (IQR) method. The first and third quartiles, Q1 and Q3, were calculated, and the interquartile range was defined IQR = Q3-Q1. Molecules with lifetimes outside the interval Q1 − 1.5×IQR to Q3 + 1.5×IQR were treated as statistical outliers. This step reduced extreme target values while preserving the main experimental variability of the dataset [17].

After target filtering, structural outliers were identified using Local Outlier Factor (LOF) and nonlinear manifold analysis [27, 28]. LOF detects anomalous molecules by comparing the local density of each compound with that of its nearest neighbors in the high-dimensional descriptor space [27]. Approximately 500 molecules, corresponding to about 7% of the dataset, were identified as LOF outliers. These molecules generally represented rare chemotypes, unusual functional-group combinations, or atypical descriptor profiles.

Uniform Manifold Approximation and Projection (UMAP) was then applied to the standardized descriptor matrix to visualize the nonlinear organization of the chemical space [28]. UMAP preserves local neighborhood relationships while revealing broader manifold structure, allowing sparse and boundary regions to be identified. Approximately 1447 molecules, corresponding to about 21% of the dataset, occupied low-density UMAP regions. These points were interpreted mainly as transitional or underrepresented chemotypes rather than necessarily erroneous data.

The intersection between LOF and UMAP outliers yielded approximately 127 molecules, representing about 1.8% of the dataset. These compounds were considered the most robust structural anomalies and were removed before final model training. The UMAP projection after outlier removal remained compact while preserving the main chemotype populations and global manifold structure. This indicates that the removed molecules were mainly isolated boundary cases rather than representative members of the dominant chromophore families.

The PCA and UMAP visualizations further demonstrate the nonlinear nature of the descriptor space. In the PCA projection shown in Fig. 5, the first two principal components capture only a limited part of the descriptor variance, and most molecules remain strongly compressed along the first component. In contrast, the UMAP representation shown in Fig. 6 reveals more clearly separated local molecular neighborhoods. This difference indicates that fluorescence-related chemical space is heterogeneous and organized through nonlinear descriptor relationships rather than simple linear variance [17].

**Fig. 4:**
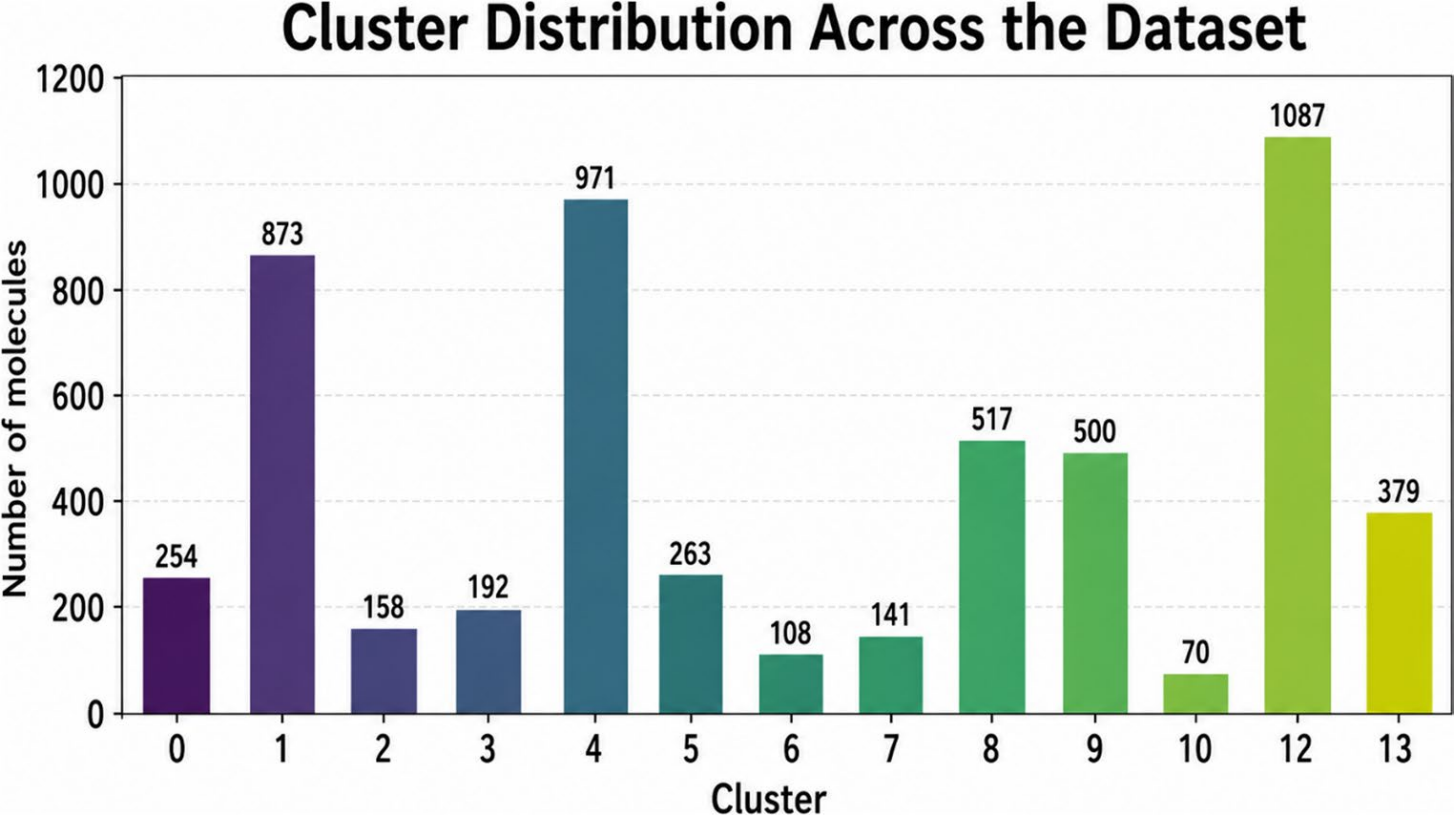
Cluster distribution across the filtered dataset. Bar plot showing the number of molecules assigned to each cluster after preprocessing and outlier removal. The distribution indicates that Clusters 12, 4, and 1 are the most populated regions of the chemical space, while smaller clusters correspond to less frequent chemotypes

**Fig. 5:**
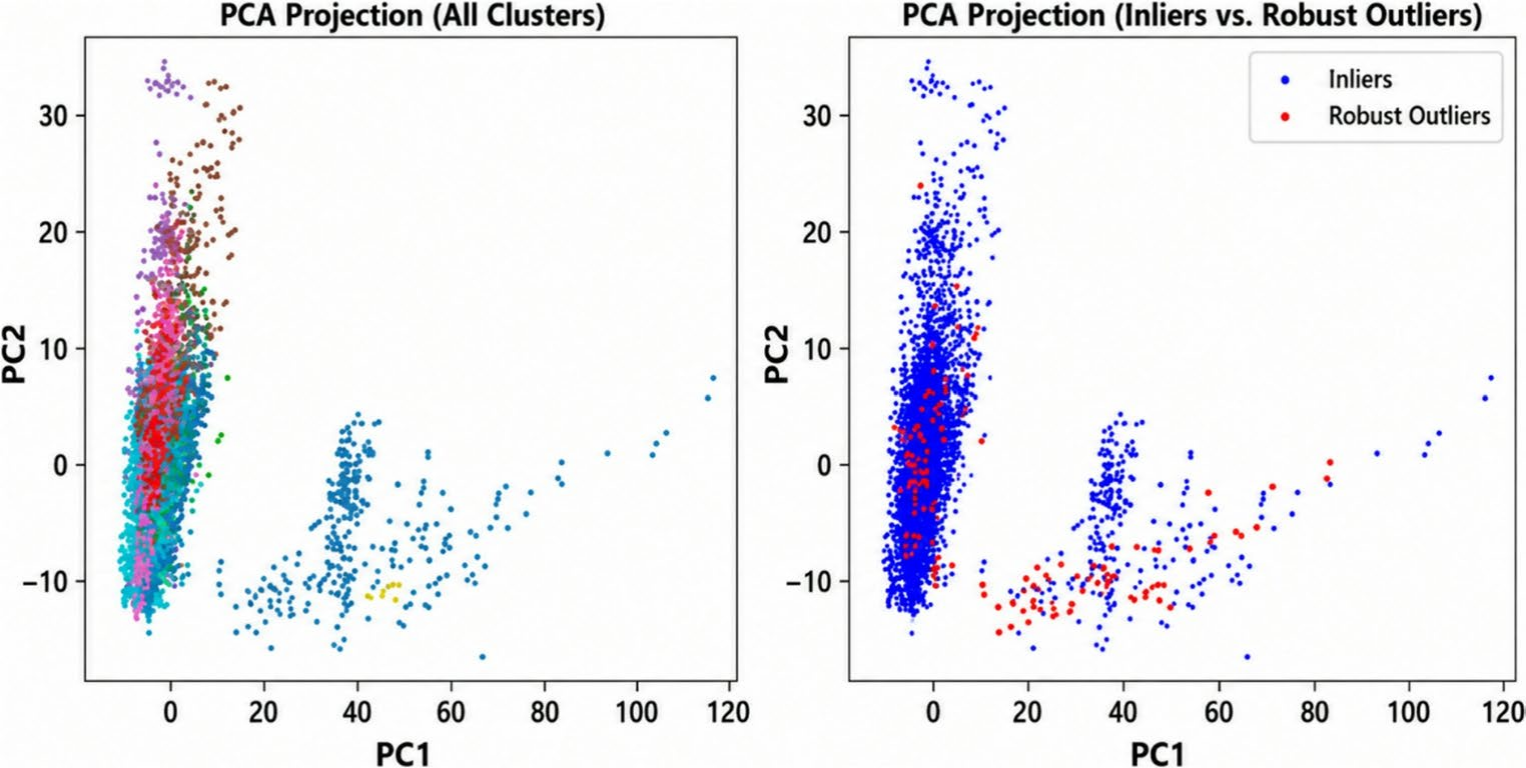
PCA visualization of cluster structure and robust outliers. Left: PCA projection colored by cluster assignment. Right: PCA projection highlighting inliers and robust outliers. The compression of most molecules along the first principal components indicates that linear dimensionality reduction captures only limited structure in the descriptor space

**Fig. 6:**
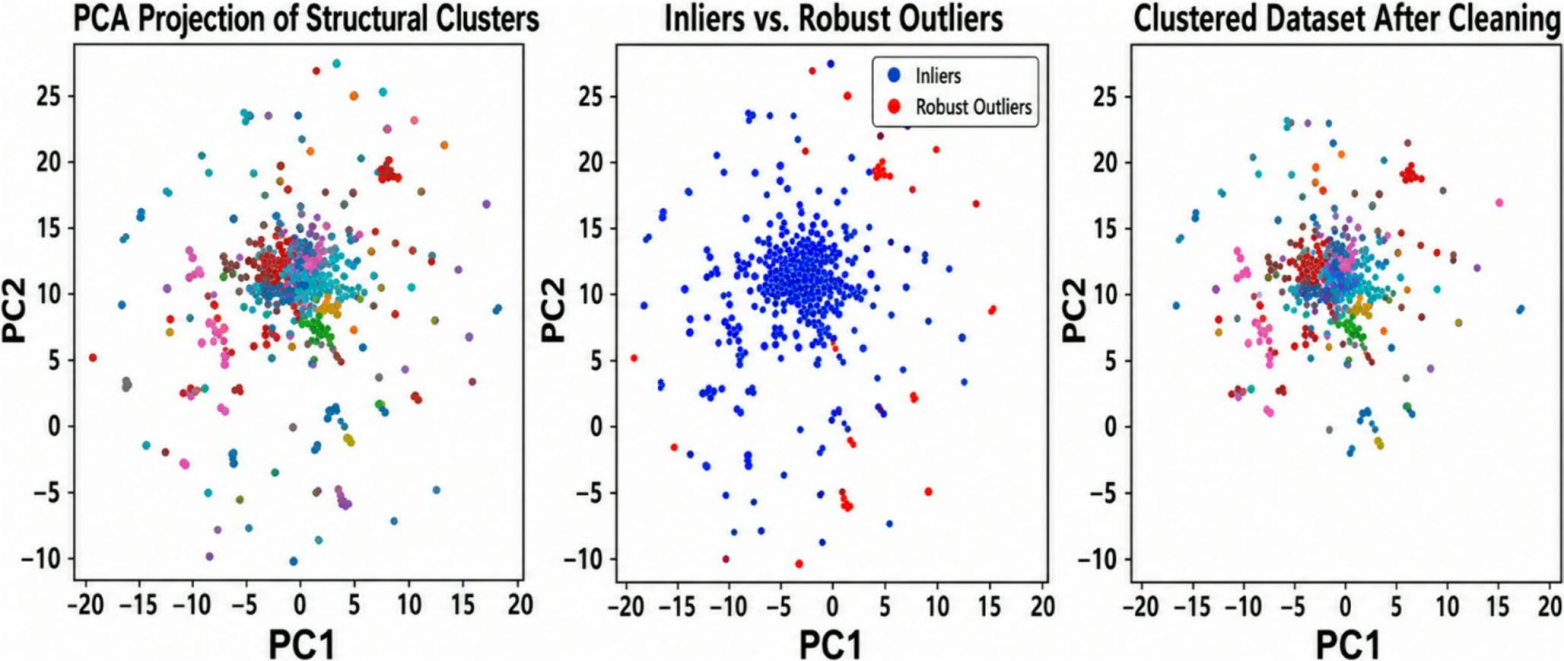
UMAP visualization of chemical space before and after robust outlier removal. Left: UMAP projection colored by cluster. Middle: robust outliers identified through the intersection of UMAP-based sparse regions and LOF anomalies. Right: UMAP projection after outlier removal, showing that the main chemical-space topology is preserved after filtering

The final cluster distribution after preprocessing is shown in Fig. 4. Several clusters remain highly populated, particularly Clusters 12, 4, and 1, indicating that aromatic and moderately conjugated chromophore families represent the dominant regions of the dataset. At the same time, smaller clusters such as 10 remain present, showing that the filtering procedure preserved rarer chemical regions. Cluster 11 disappeared after robust outlier removal, suggesting that it mainly consisted of structurally isolated molecules identified as anomalous by both LOF and UMAP. Overall, the preprocessing strategy improved dataset consistency while retaining broad chemical-space diversity required for reliable model generalization Figs. 5 and 6.

Following outlier removal, both the target variable and the input descriptors were transformed to reduce skewness and improve numerical stability. The fluorescence lifetime values were transformed using a log(1 + x) function, which compresses large lifetime values while preserving near-zero values. This step reduced the dominance of long-lived fluorophores during training. In parallel, a PowerTransformer was applied to the descriptor matrix to stabilize variance, reduce the influence of extreme descriptor values, and improve model generalization [17]. These transformations produced a more balanced numerical representation for gradient-boosted decision tree learning [15].

### Model Development

After preprocessing, approximately 5000 chromophore–solvent entries were retained for downstream modeling. To further reduce noise and dimensionality, low-variance descriptors were removed, since features with minimal variation across the dataset contribute little predictive information. Recursive feature elimination (RFE) was then applied to identify the most informative descriptors for fluorescence lifetime prediction [17]. RFE iteratively trains a model, ranks descriptors according to their importance, and removes the least relevant features until an optimal subset is obtained.

**Fig. 7:**
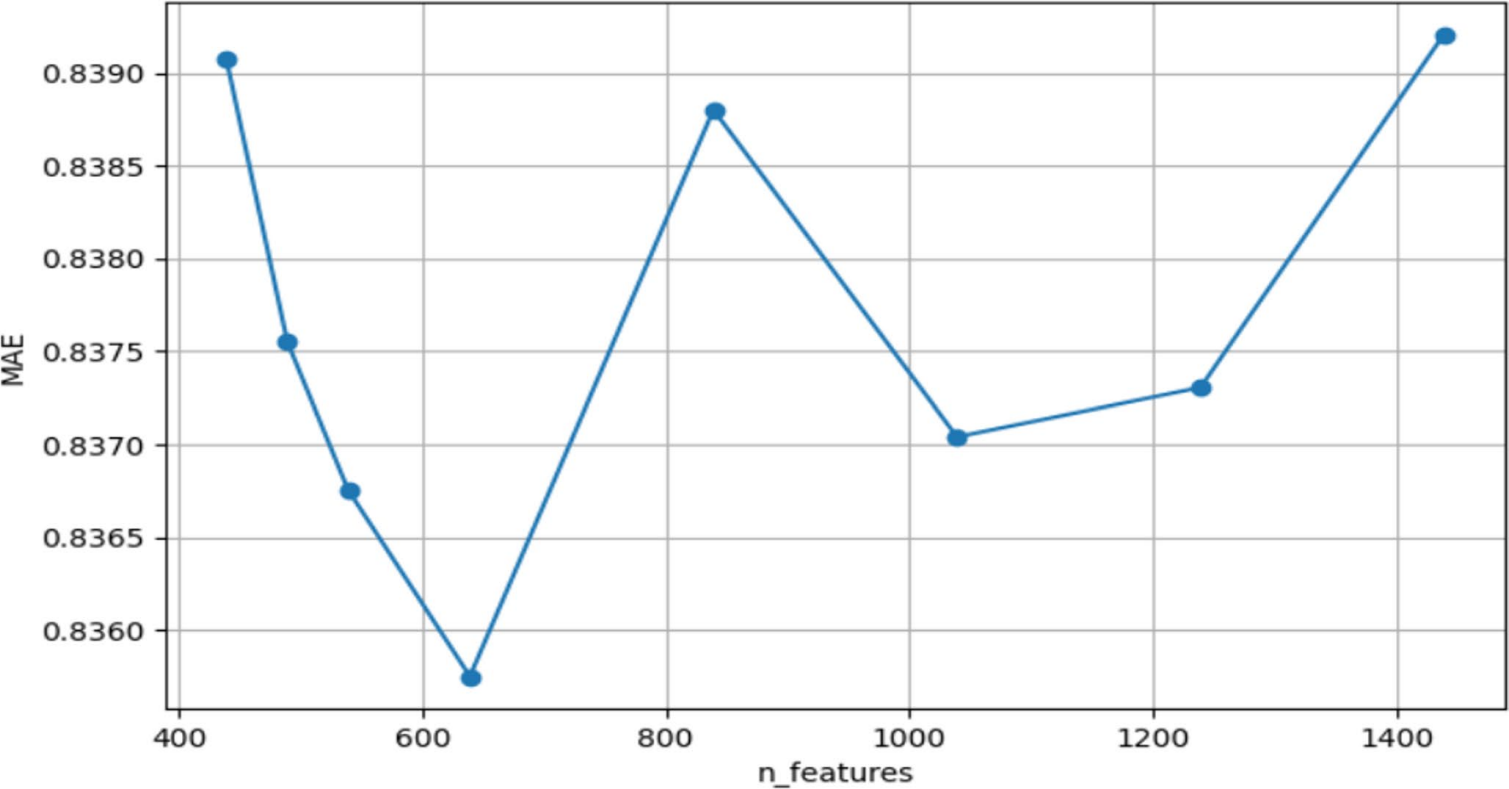
Influence of feature number on model prediction error Variation of mean absolute error (MAE) as a function of the number of selected descriptors during recursive feature elimination. The curve shows that model performance remains relatively stable over a broad feature range, with the lowest MAE obtained near the selected feature subset, indicating that redundant descriptors can be removed without substantially degrading predictive accuracy

This feature-selection step reduced computational cost, improved model interpretability, and lowered the risk of overfitting. These benefits are particularly important for high-dimensional cheminformatics datasets, where many descriptors may be redundant or weakly informative. As shown in Fig. 7, the mean absolute error (MAE) [17] remained relatively stable across a broad range of selected feature numbers, indicating that many descriptors carried overlapping information. The lowest MAE was obtained around the selected feature subset, supporting the use of a reduced descriptor space without substantial loss of predictive performance.

The reduced feature set also improves the maintainability of the workflow. Future descriptors can be added and re-evaluated using the same feature-selection procedure, allowing the model to be expanded while retaining only variables with clear predictive value.

However, excessive feature removal may lead to the loss of important chemical information and reduce model performance. Therefore, the final feature subset was selected as a compromise between minimal prediction error, reduced dimensionality, and chemical interpretability.

To train and evaluate the model, a 10-fold stratified cross-validation strategy was used, with the cluster label serving as the stratification variable [17]. This ensured that each fold contained a balanced representation of the main structural and physicochemical molecular groups, reducing bias caused by overrepresented chemotypes. Hyperparameter optimization was performed with Optuna [29] tuning parameters such as learning rate, maximum tree depth, number of leaves, feature fraction, and bagging fraction. This procedure allowed systematic refinement of the LightGBM model [15] and improved predictive stability across validation folds.

Because the cluster distribution was imbalanced, stratification was essential to avoid training or validation folds dominated by the largest molecular families. By preserving the relative distribution of clusters across folds, the evaluation better reflected the chemical diversity of the dataset and reduced variance in the reported performance metrics. This strategy was particularly important for fluorescence lifetime prediction, where model accuracy can depend strongly on the structural composition of the validation set.

To support reproducibility and future reuse, the trained LightGBM model and the associated preprocessing pipeline is deposited in a public repository GitHub provided in the Supplementary data file. Providing the model together with descriptor-generation scripts, preprocessing parameters, and feature-selection information would allow other researchers to apply the workflow to new chromophores without retraining, while also ensuring long-term accessibility and transparency of the computational procedure.

## Results and Discussion

The metrics used for model evaluation were MAE and R² score [17]. MAE represents the mean absolute error, providing a direct measure of predictive accuracy, while R² indicates the proportion of variance in the dataset explained by the model.

2 $$\:MAE=\frac{1}{N}{\sum\:}_{k=1}^{N}\left|{\widehat{y}}_{i}-{y}_{i}\right|$$

3 $$\:{R}^{2}=1-\frac{{\sum\:}_{k=1}^{N}{\left({\widehat{y}}_{i}-{y}_{i}\right)}^{2}}{{\sum\:}_{k=1}^{N}\left(\stackrel{-}{y}-{y}_{i}\right)}$$

where $\:{\widehat{y}}_{i}$ is the predicted value and $\:{y}_{i}$ true value for the i-th entry and $\:\stackrel{-}{\mathrm{y}}$ is the mean value of the true values.

Using the optimized LightGBM model, the stratified cross-validation procedure yielded a mean MAE of 0.8324 ± 0.0617 ns and a mean R² of 0.7523 ± 0.0317, indicating stable predictive behavior across folds. The best-performing fold achieved MAE = 0.712 ns and R² = 0.806, demonstrating that under favorable train–validation partitioning the model can capture a larger fraction of the fluorescence lifetime variability. The fold-by-fold metrics and optimized hyperparameters are reported in Supplement Information file.

The difference between the average cross-validation performance and the best-performing fold reflects the chemical heterogeneity of the dataset. Fluorescence lifetime depends on nonlinear and context-dependent interactions between chromophore structure, solvent environment, molecular rigidity, polarity, and photophysical descriptors [1, 2]. Therefore, model performance can vary depending on the structural composition of each validation fold. This behavior supports the use of cluster-stratified cross-validation, which helps maintain balanced chemotype representation across folds and reduces bias caused by structurally uneven data splits [22].

**Fig. 8:**
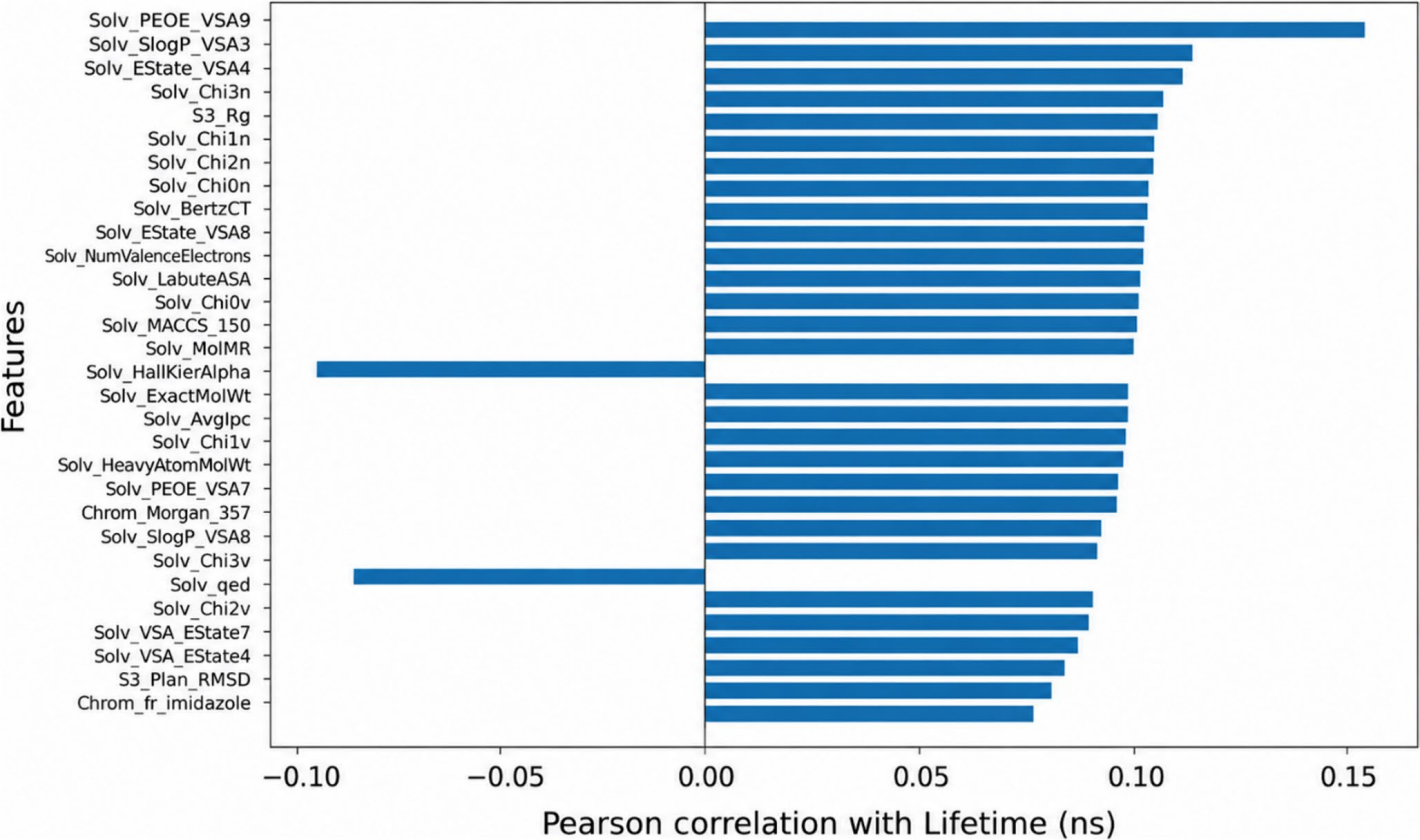
Pearson correlation coefficients with the ‘Lifetime (ns)’

Pearson correlation analysis [17] further confirms the nonlinear nature of the prediction problem presented in Fig. 8. Most individual descriptors showed relatively weak linear correlation with fluorescence lifetime, indicating that the target property is not controlled by simple one-variable relationships. Instead, lifetime emerges from higher-order interactions between molecular topology, electronic structure, solvent effects, and experimental photophysical variables. Therefore, low Pearson correlation values do not imply that the descriptors are uninformative; rather, their predictive value becomes apparent through nonlinear combinations captured by ensemble models such as LightGBM [15].

**Fig. 9:**
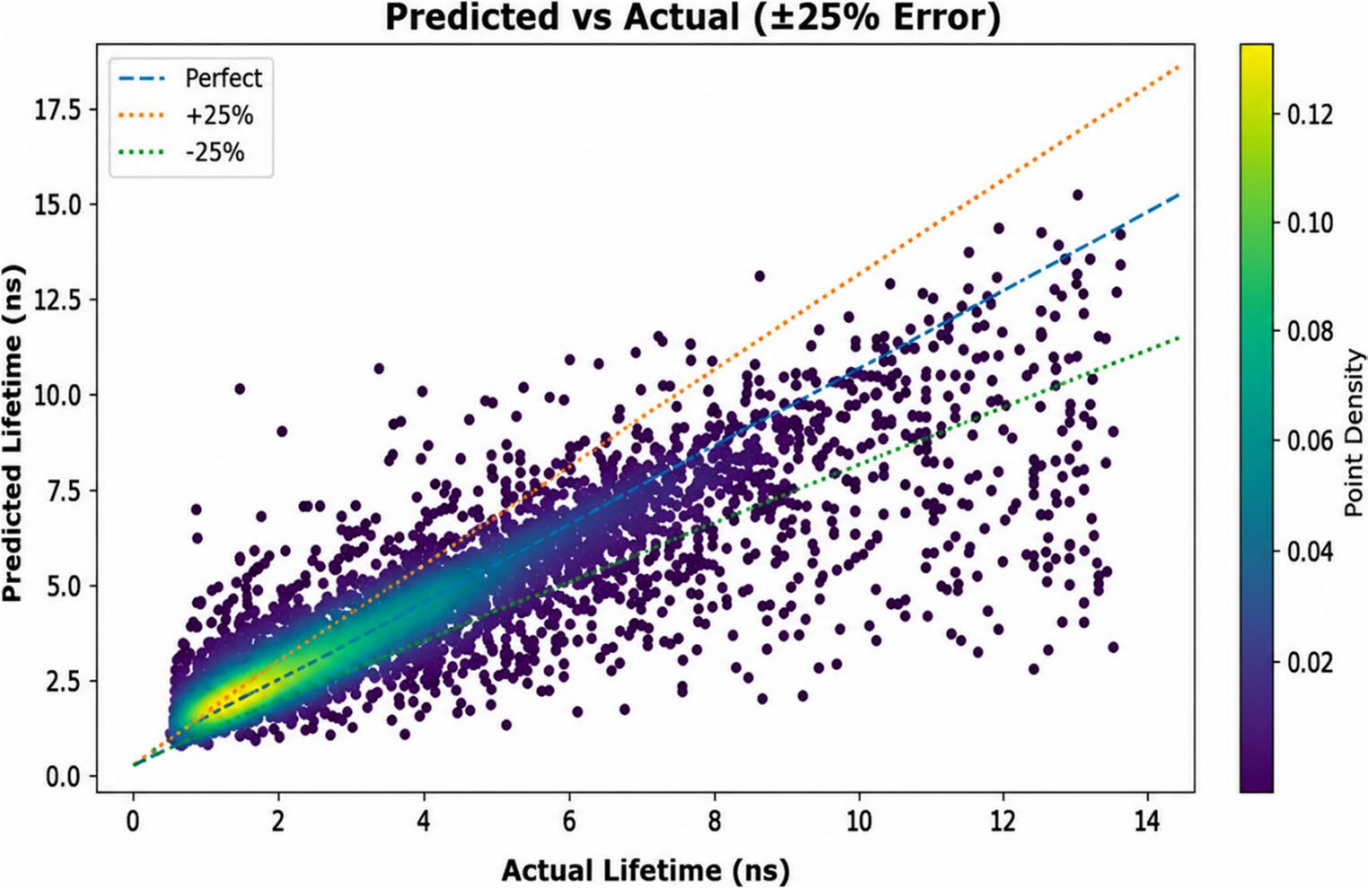
Predicted versus experimental fluorescence lifetimes. Scatter plot comparing LightGBM-predicted fluorescence lifetimes with experimentally measured values. The dashed blue line represents perfect agreement, while the dotted lines indicate ± 25% relative error. Point color represents local density. The highest density occurs near short and intermediate lifetimes, where most predictions remain close to the ideal line, whereas larger deviations are observed for long-lifetime chromophores

The prediction plot shown in Fig. 9 demonstrates that the model captures the global trend of the data. Most points are concentrated near the ideal prediction line, especially in the high-density region corresponding to short and intermediate lifetimes. A large fraction of predictions falls within or close to the ± 25% error margins, indicating reliable performance within the main applicability domain of the dataset. However, the spread increases at longer lifetimes, where the model tends to show larger deviations and partial regression toward the central lifetime range. This behavior is consistent with the lower data density and greater chemical diversity observed for long-lived chromophores.

To assess model interpretability, SHapley Additive exPlanations (SHAP) were used to quantify the contribution of each descriptor to the predicted fluorescence lifetime [12]. SHAP is a game-theoretic method that assigns an importance value to each feature for each individual prediction, indicating both the magnitude and direction of its effect. Positive SHAP values increase the predicted lifetime, whereas negative values decrease it. This approach is particularly useful for complex ensemble models such as LightGBM, where nonlinear interactions between many molecular, solvent, and photophysical descriptors can be difficult to interpret directly.

**Fig. 10:**
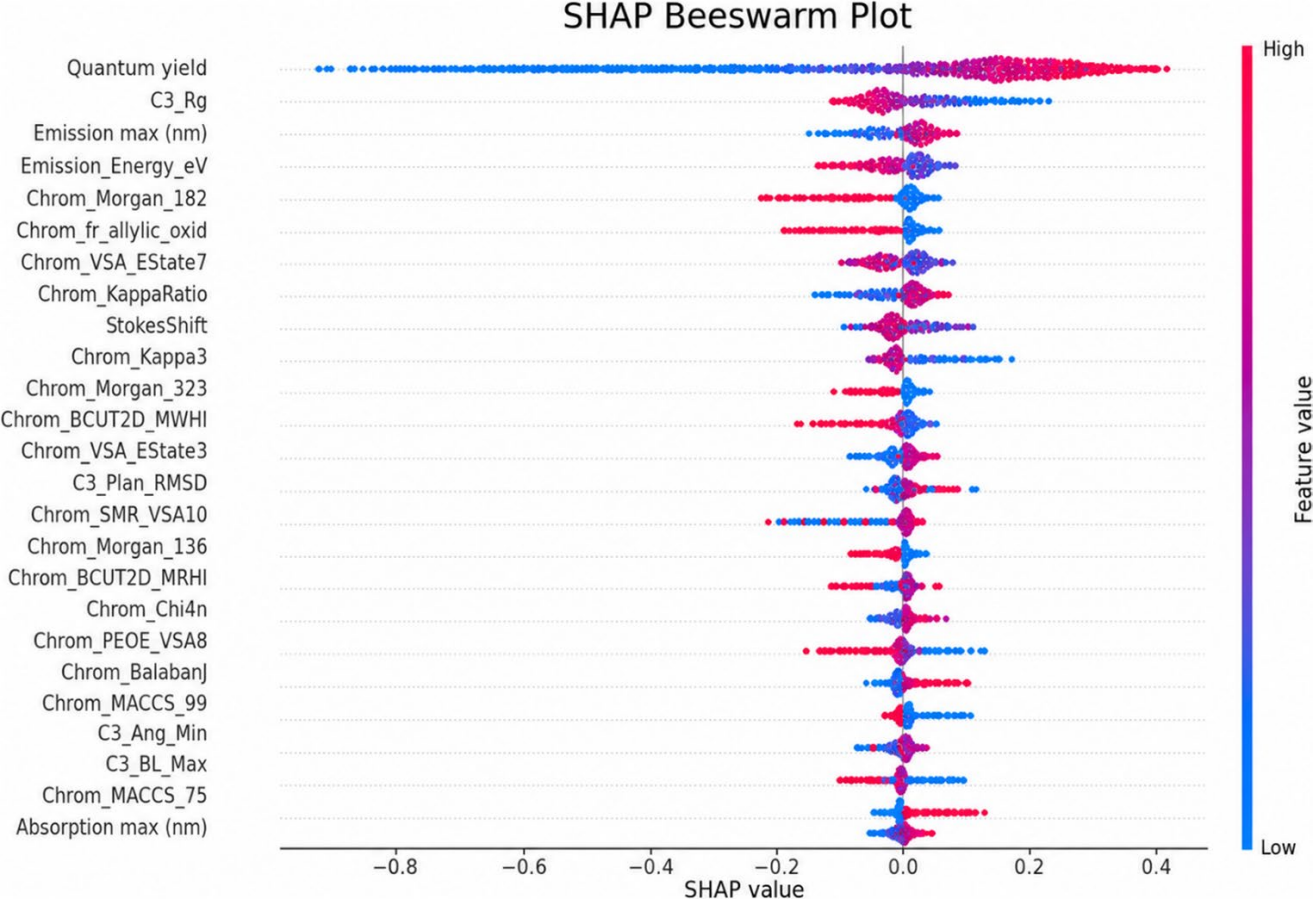
Global SHAP analysis of the fluorescence lifetime prediction model. SHAP summary plot showing the most influential descriptors ranked by their mean absolute contribution to the LightGBM model output. Each point represents one molecule, with positive SHAP values (red) increasing the predicted lifetime and negative values (blue) decreasing it. The plot identifies the molecular, solvent, and photophysical descriptors most strongly associated with fluorescence lifetime prediction

SHAP values were calculated for all molecules in the final dataset. The SHAP summary plot shown in Fig. 10 therefore represents the global behavior of the model across the studied chemical space. Features are ranked according to their overall contribution to model output, while the distribution of SHAP values shows how each descriptor affects predictions across different molecules. The results indicate that a relatively small group of descriptors accounts for most of the model interpretability, highlighting the key structural, electronic, and environmental factors associated with fluorescence lifetime modulation.

Among the most influential descriptors identified by SHAP analysis, quantum yield emerged as the dominant contributor to fluorescence lifetime prediction, as shown in Fig. 10. This result is physically consistent with the relationship between fluorescence lifetime and the competition between radiative and non-radiative decay pathways [1, 2]. Molecules with high quantum yield values generally show positive SHAP contributions, indicating longer predicted lifetimes, whereas low quantum yield values are associated with negative SHAP contributions and shorter predicted lifetimes. This confirms that the model captures the expected link between radiative efficiency and excited-state persistence.

Fluorescence lifetime is governed by the total decay rate:

4 $$\:\tau\:=\frac{1}{{k}_{r}+{k}_{nr}}$$

and the fluorescence quantum yield is defined as:

5 $$\:{\Phi\:}=\frac{{k}_{r}}{{k}_{r}+{k}_{nr}}={k}_{r}\tau\:$$

where k_r_ is the radiative decay rate, k_nr_ is the non-radiative decay rate, τ is the fluorescence lifetime, and Φ is the fluorescence quantum yield. Therefore, high quantum yield generally indicates that radiative decay is favored relative to non-radiative relaxation, explaining its strong positive influence on lifetime prediction.

The chromophore radius of gyration, represented as C3_Rg in Fig. 10, was the most important geometry-related descriptor. This descriptor measures the spatial distribution of atoms around the molecular center and reflects whether the chromophore is compact or extended [22]. The mass-weighted center of mass and radius of gyration are defined as:

6 $$\:{r}_{cm}=\frac{{\sum\:}_{i=1}^{N}{m}_{i}{r}_{i}}{{\sum\:}_{i=1}^{N}{m}_{i}}$$

7 $$\:{R}_{g}=\sqrt{\frac{{\sum\:}_{i=1}^{N}{m}_{i}{\left|{r}_{i}-{r}_{cm}\right|}^{2}}{{\sum\:}_{i=1}^{N}{m}_{i}}}$$

where m_i_ and r_i_ are the mass and position vector of atom i, respectively. In the SHAP plot, high C3_Rg values are mainly associated with negative SHAP contributions, suggesting that more spatially extended chromophores tend to have shorter predicted lifetimes. This may reflect increased conformational flexibility, solvent exposure, and low-frequency vibrational modes, all of which can promote internal conversion and non-radiative energy loss.

Emission-related descriptors, including Emission max (nm), Emission_Energy_eV, and StokesShift, also ranked among the most influential features. Their importance indicates that lifetime is closely connected to the excited-state energy landscape, including electronic delocalization, solvent-induced stabilization, and relaxation after excitation. In Fig. 10, longer emission wavelengths generally contribute positively to lifetime prediction, while higher emission energies show the opposite tendency because emission energy is inversely related to wavelength. The importance of Stokes shift further suggests that geometric relaxation, solvent reorganization, and charge-transfer character contribute to lifetime modulation.

The engineered descriptor Chrom_KappaRatio was introduced to describe the balance between molecular linearity and higher-order topological complexity:

8 $$\:ChromkappaRatio=\frac{{\kappa\:}_{1}}{{\kappa\:}_{2}+{\kappa\:}_{3}+{10}^{-6}}$$

This descriptor combines Kier molecular shape indices, where **κ₁** mainly reflects molecular elongation, while κ₂ and κ₃ encode increasing levels of branching, cyclicity, and global topological complexity [21]. High Chrom_KappaRatio values are associated with more ordered and extended molecular shapes, which can support continuous π-conjugation and excited-state stabilization. In contrast, lower values may indicate increased branching, non-planarity, or disrupted conjugation, which can enhance vibrational relaxation and shorten fluorescence lifetime.

Among the individual Kappa indices, Chrom_Kappa3 was also important. Higher Chrom_Kappa3 values tend to contribute negatively or near-neutrally in Fig. 10, whereas lower values are more often associated with positive SHAP contributions. This suggests that increased topological complexity is generally unfavorable for long fluorescence lifetimes. Structurally, such complexity may arise from branched frameworks, fused aromatic systems, anisotropic geometries, or non-uniform arrangements of conjugated fragments. These features can disturb efficient electronic delocalization and increase conformational relaxation pathways.

Several fragment-level descriptors further refine this interpretation. Chrom_VSA_EState7 and Chrom_VSA_EState3 describe electrotopological-state contributions distributed over van der Waals surface areas, combining information about local polarity, substituent environment, and molecular surface exposure [18]. Their importance suggests that weakly or moderately polar surface fragments can influence lifetime by modifying local electron density, charge-transfer character, and vibronic coupling.

Other descriptors, including Chrom_SMR_VSA10, Chrom_BCUT2D_MWHI, and Chrom_BCUT2D_MRHI, provide information about steric effects, molar refractivity, polarizability, and atomic-property distributions [18]. Their SHAP behavior indicates that steric restriction, local polarity, and heavy-atom or high-mass environments can influence excited-state stability. In particular, high Chrom_BCUT2D_MWHI values tend to contribute negatively, suggesting that heavy-atom-rich or high-mass atomic environments may enhance non-radiative pathways in some structural contexts.

**Fig. 11:**
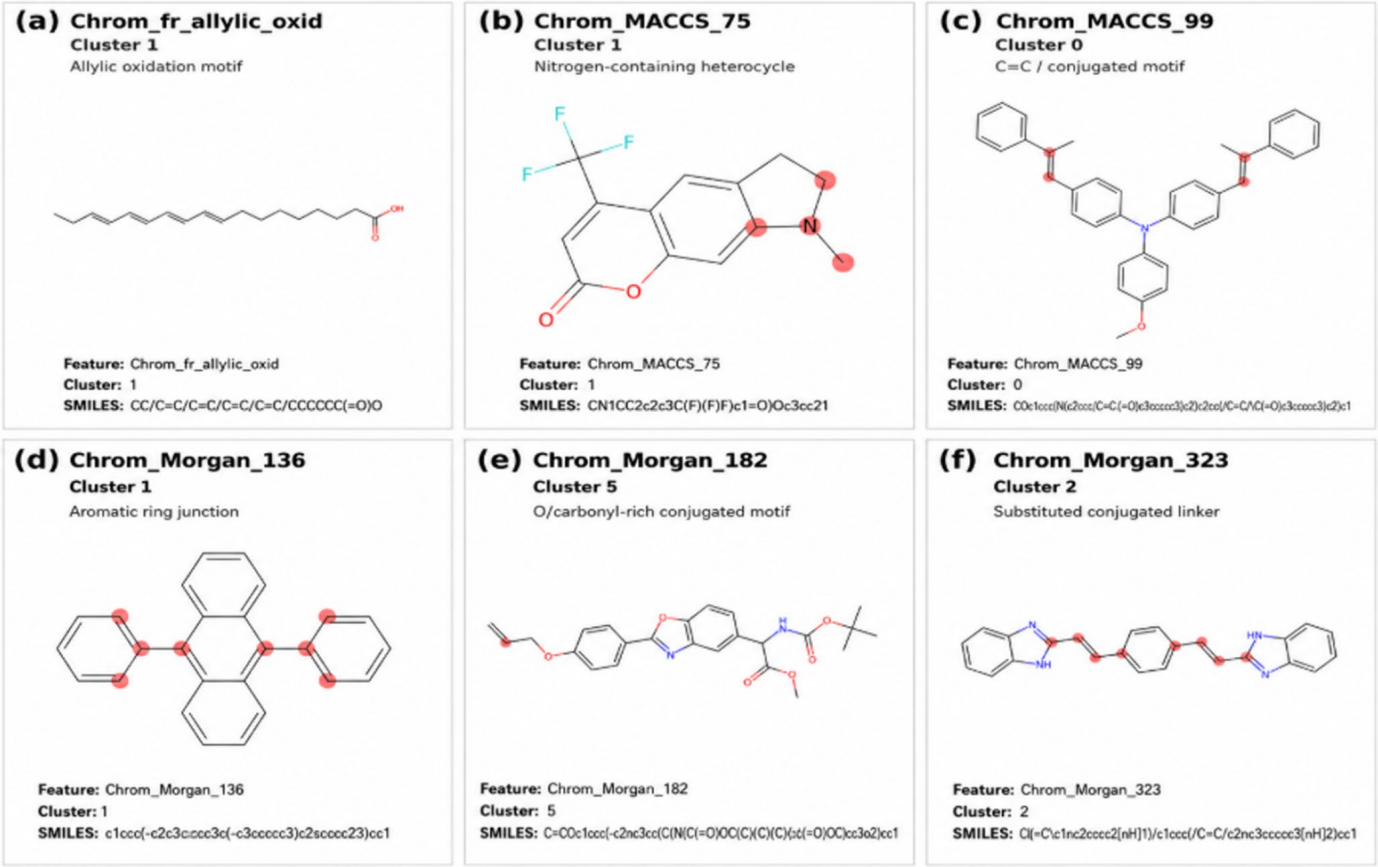
Representative molecular fragments associated with important SHAP descriptors

The descriptor Chrom_fr_allylic_oxid was also identified as an important contributor to fluorescence lifetime prediction. This descriptor captures allylic oxidation-related motifs, typically involving carbon atoms adjacent to double bonds or conjugated systems connected to oxygen-containing functionalities. A representative example is shown in Fig. 11a, where the motif appears in an unsaturated flexible chain containing an oxygenated terminal group. In the SHAP analysis, high values of Chrom_fr_allylic_oxid are associated with shorter predicted lifetimes, suggesting that these motifs promote faster excited-state deactivation. This can be attributed to localized lone-pair orbitals, possible n→π* character, conformational flexibility, and additional vibrational relaxation pathways, all of which may enhance internal conversion and non-radiative decay.

The MACCS descriptors provide complementary chemically interpretable information. Chrom_MACCS_75, shown in Fig. 11b, corresponds to a nitrogen-containing heterocyclic environment. Its effect should be interpreted as context-dependent: heteroatoms may promote lone-pair-driven relaxation, but when embedded in rigid aromatic or heterocyclic frameworks, they can also contribute to electronic stabilization and conformational restriction. Therefore, this descriptor should not be considered universally quenching, but rather as a motif whose influence depends on the surrounding molecular architecture.

Chrom_MACCS_99, illustrated in Fig. 11c, represents a carbon–carbon double-bond or unsaturated structural motif. Such π-containing environments are central to fluorescence because they contribute to electronic delocalization and excited-state stabilization. Their influence on lifetime depends strongly on whether the unsaturated motif is part of a rigid conjugated scaffold or a more flexible substituted framework. This supports the broader conclusion that conjugation favors longer lifetimes only when accompanied by structural rigidity and preserved orbital overlap.

The Morgan fingerprint descriptors provide more localized atom-centered structural information. Chrom_Morgan_136, shown in Fig. 11d, highlights aromatic ring-junction environments within an extended polycyclic scaffold. This motif reflects rigid π-conjugated regions that can stabilize excited states. However, its contribution is not purely positive, indicating that large aromatic systems may also introduce size effects, substituent-dependent relaxation pathways, or additional non-radiative channels depending on the broader molecular context.

**Fig. 12:**
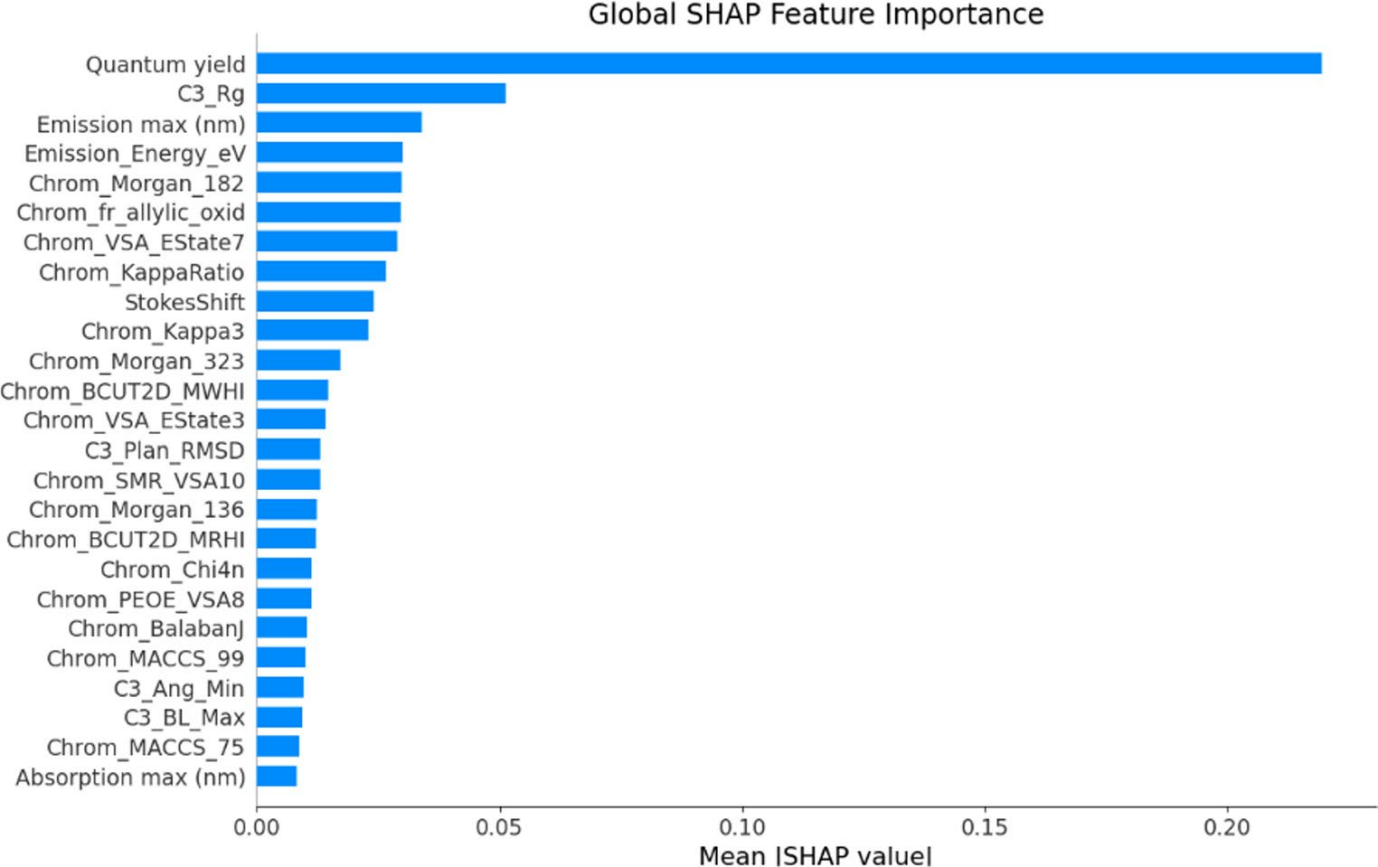
Global SHAP feature importance for the fluorescence lifetime model. Mean absolute SHAP values of the most influential descriptors in the LightGBM model. The ranking shows that quantum yield is the dominant predictor, followed by geometry-related, emission-related, topological, and fragment-level descriptors

Chrom_Morgan_182 was one of the most important fingerprint descriptors in the global SHAP ranking shown in Fig. 12. The representative structure in Fig. 11e highlights a conjugated heteroatom-containing environment involving oxygen- and carbonyl-rich functionality. This descriptor is particularly relevant because Souza et al. [10] previously identified Morgan_182 as an important chromophore fingerprint for fluorescence quantum yield prediction. In their work, this bit was associated with a double-bond-containing substructure contributing to electronic conjugation and fluorescence efficiency.

In the present lifetime model, however, high values of Chrom_Morgan_182 are mainly associated with shorter predicted lifetimes. This does not contradict the interpretation of Souza et al. [10], but reflects the different physical meaning of fluorescence lifetime compared with quantum yield or emission wavelength. While conjugated double-bond environments can improve radiative efficiency, the local topology encoded by Chrom_Morgan_182 in this dataset often appears coupled to polar oxygen-containing groups, carbonyl fragments, or flexible internally substituted environments. These features can enhance vibronic coupling, solvent interaction, and non-radiative relaxation, leading to shorter lifetimes.

Chrom_Morgan_323, shown in Fig. 11f, highlights an internally substituted conjugated linker connecting heteroaromatic fragments. In the SHAP analysis, high values of this descriptor are mainly associated with shorter predicted lifetimes. Although the motif contains π-conjugated character, the internal substitution pattern may introduce torsional flexibility, geometric relaxation, or partial disruption of conjugation continuity. These effects can increase electronic-vibrational coupling and favor internal conversion. Thus, Chrom_Morgan_323 represents a case where conjugation alone does not necessarily lead to longer lifetime because local flexibility and substitution effects may promote non-radiative relaxation.

Overall, the SHAP analysis in Fig. 10 and the representative molecular fragments in Fig. 11 support a physically coherent interpretation of the model. Longer fluorescence lifetimes are favored by high quantum yield, compact molecular geometry, stabilized lower-energy emissive states, and ordered conjugated topologies. In contrast, shorter lifetimes are associated with extended or flexible geometries, high topological complexity, allylic oxidation motifs, polar oxygen/carbonyl-containing environments, substituted unsaturated linkers, and local structural motifs that enhance non-radiative relaxation. These results indicate that the model captures chemically meaningful relationships between molecular structure, excited-state stabilization, and fluorescence lifetime behavior rather than relying only on statistical correlations.

Despite the overall robustness of the developed model, several limitations remain. The model does not explicitly include experimental conditions such as pH, temperature, ionic strength, oxygen concentration, viscosity, or detailed solvent microenvironment effects. These parameters can influence fluorescence lifetimes by modifying protonation states, hydrogen-bonding networks, intermolecular interactions, and the balance between radiative and non-radiative decay pathways. Therefore, part of the residual prediction error may originate from experimental variables that are not encoded in the current descriptor set. This limitation is especially relevant for acidic, basic, or strongly solvatochromic chromophores, where small environmental changes can substantially alter excited-state behavior.

The predicted-versus-actual plot shown in Fig. 9 demonstrates that the model captures the global lifetime trend well. The highest point density is concentrated close to the ideal prediction line, particularly in the short- and intermediate-lifetime regions. Most molecules fall within or near the ± 25% error boundaries, indicating reliable prediction performance within the main applicability domain of the dataset. However, the spread increases for longer lifetimes, where the model shows larger deviations from the ideal line. This suggests that the model learns dominant structure–property relationships effectively in densely populated regions of chemical space, but becomes less precise for sparsely represented long-lifetime chromophores. Figure 12 presents the most meaningful descriptors in terms of Mean Shap value [12].

**Fig. 13:**
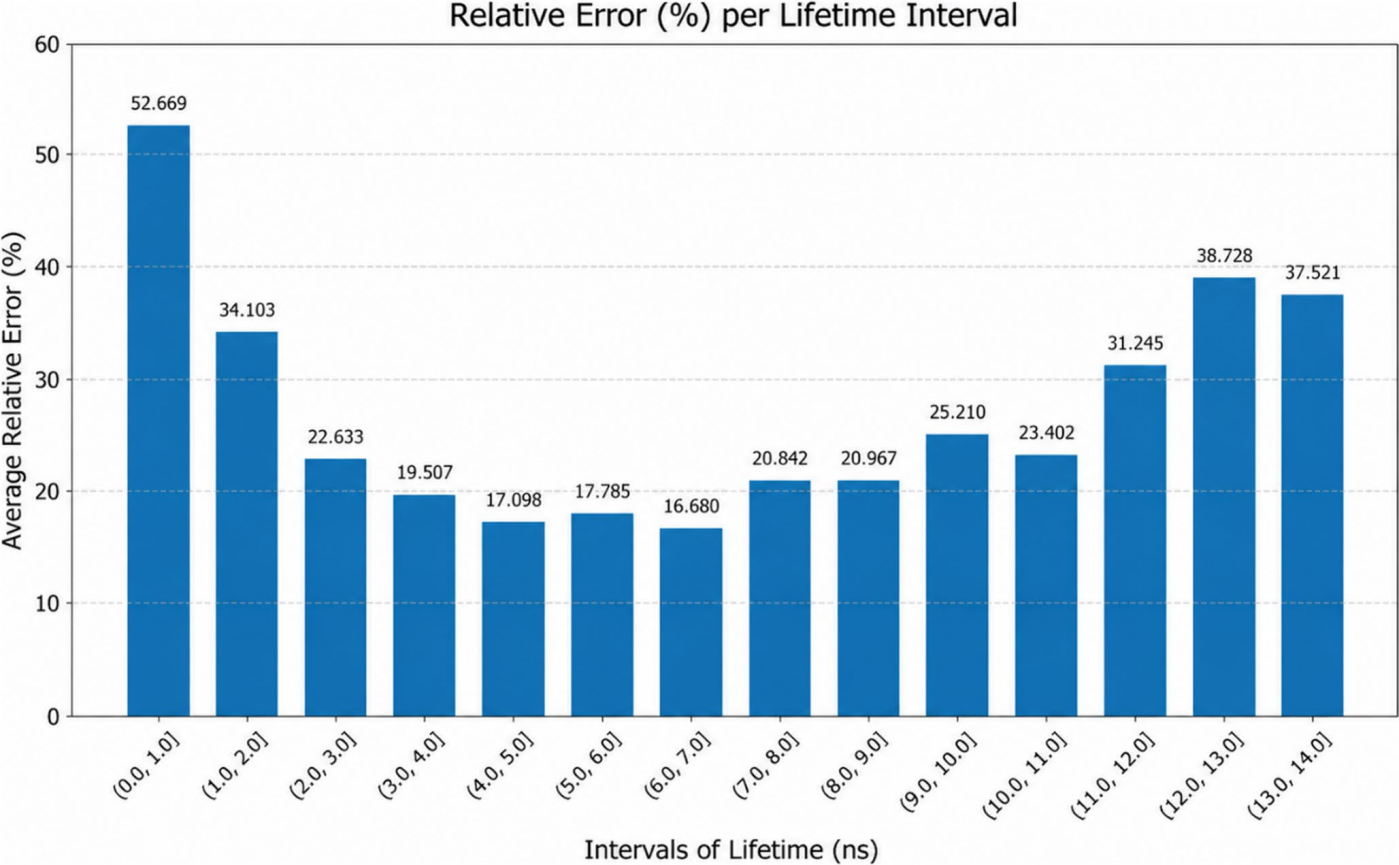
Relative prediction error across fluorescence lifetime intervals. Average relative error (%) calculated for different experimental lifetime intervals. The model shows the highest relative error in the sub-nanosecond region, where percentage error is strongly amplified by small lifetime values

The interval-based relative error analysis shown in Fig. 13 confirms that model performance depends strongly on the lifetime regime. The highest average relative error occurs in the sub-nanosecond interval, where small absolute deviations produce large percentage errors. The error then decreases and remains more stable across the intermediate lifetime range, particularly between approximately 3 and 10 ns, indicating that this region represents the most reliable prediction domain of the model. At longer lifetimes, especially above 10 ns, the relative error increases again, most likely because long-lived chromophores are less frequent in the training data. This behavior indicates that the model tends to perform best in well-populated lifetime regions and becomes less reliable near the extremes of the distribution.

The interval-based relative error analysis shown in Fig. 13 further confirms that model performance is regime-dependent rather than uniformly distributed across the full lifetime range. The highest relative error is observed in the sub-nanosecond region, especially below 1 ns. This behavior should be interpreted carefully because very small lifetime values amplify percentage error: even a small absolute difference between measured and predicted values can produce a large relative deviation. In addition, sub-nanosecond lifetimes are experimentally more sensitive to instrumental resolution, detector response, and photon-counting uncertainty, making this region intrinsically more difficult to model.

In the intermediate lifetime region, approximately between 3 and 10 ns, the relative error remains lower and more stable. This range corresponds to the densest and most statistically regular portion of the dataset, where both experimental measurements and descriptor–target relationships are better represented. Therefore, the model is most reliable in this interval, which likely represents the main applicability domain of the current training data. At longer lifetimes, particularly above 10 ns, the error increases again. Unlike the sub-nanosecond region, this trend is mainly attributed to data sparsity rather than percentage-error inflation. Long-lifetime chromophores are less frequent in the dataset, so the model has fewer representative examples from which to learn this behavior. As a result, predictions in this region tend to regress toward the dominant central lifetime distribution [6].

Overall, Figs. 9 and 13 show that the model captures the main fluorescence lifetime trend well, but remains less reliable at the extremes of the lifetime distribution. The short-lifetime regime is affected by relative-error inflation and possible experimental uncertainty, whereas the long-lifetime regime is mainly limited by insufficient sample representation. These limitations do not indicate a general failure of the model, but rather define its current applicability domain.

To further evaluate generalizability, two external validation strategies were considered. First, model predictions were compared with the TD-DFT fluorescence lifetime benchmark reported by Wong et al., who evaluated 24 fluorophores using several functionals and lifetime-calculation schemes [14]. To avoid overlap with the training dataset, only chromophore–solvent pairs not present in training dataset were retained. The resulting comparison is summarized in Table S8 from the Supplementary Data, where ML predictions are reported alongside experimental lifetimes and TD-DFT Scheme C errors obtained with BMK, LC-BLYP*, and ωB97X*. For this non-overlapping subset, the ML model achieved an MAE of 1.694 ns and a mean absolute percentage error of 30.50%. The TD-DFT methods performed slightly better on this curated benchmark, with MAE values of 1.485 ns for BMK, 1.407 ns for LC-BLYP*, and 1.182 ns for ωB97X*. Nevertheless, the ML model produced competitive estimates for many fused aromatic, acridone, bimane, and coumarin systems, especially in the intermediate lifetime range.

The TD-DFT comparison also highlights the complementary nature of the two approaches. TD-DFT provides a more theoretical method for determining radiative transitions, excited-state relaxation, and vibronic effects, but its accuracy depends on the selected functional, solvent model, and treatment of non-radiative processes [13]. The ML model, in contrast, learns directly from experimental structure–property relationships and offers rapid predictions at much lower computational cost. Therefore, discrepancies between ML and TD-DFT should not be interpreted solely as model failure, since theoretical lifetimes also contain methodological approximations. The most relevant benchmark remains agreement with experimental measurements, even though experimental data also contain uncertainty from sample preparation, instrumental response, and environmental variability.

Second, the model was tested against an additional external experimental set containing chemically diverse chromophores not used in model development. These molecules included perylene derivatives, cyanine and merocyanine dyes, BODIPY-type systems, coumarins, benzoxazole derivatives, flavin-like structures, and donor–acceptor oligomers. The results are summarized in Table S7 from Supplementary Data together with their references. The strongest agreement is observed for several benzoxazole derivatives, BODIPY systems, methyl perylene carboxylate, carbazole–iodo-BODIPY, and medium-lifetime donor–acceptor dyes.

Together, the TD-DFT and external experimental validations show that the model is most useful as a fast screening and prioritization tool rather than a replacement for high-precision spectroscopy or detailed quantum-chemical calculations. Its main strength lies in capturing global structure–property trends across broad chemical space, especially within well-represented lifetime regimes. Larger deviations occur for very short-lived chromophores, where relative error is amplified, and for long-lived or structurally unusual systems, where training examples are sparse. Therefore, the model should be interpreted as a low-cost surrogate for identifying promising candidates and guiding further experimental or TD-DFT investigation.

## Conclusions

In summary, this study demonstrates that a LightGBM-based machine learning framework can effectively model fluorescence lifetimes of organic chromophores using structural, electronic, photophysical, and solvent-dependent descriptors. By combining the Deep4Chem dataset with descriptor engineering and feature selection, the model achieved stable cross-validation performance, with a mean MAE of 0.8324 ± 0.0617 ns and a mean R² of 0.7523 ± 0.0317. These results show that gradient boosting methods provide an efficient and interpretable strategy for fluorescence lifetime estimation, particularly for high-throughput screening applications where experimental measurements or quantum-chemical calculations are time-consuming.

Beyond prediction accuracy, SHAP analysis provided insight into the molecular factors controlling fluorescence lifetime. Quantum yield emerged as the dominant descriptor, consistent with its direct relationship and correlation with the theory. Additional structural and electronic descriptors, including molecular geometry, topology, rigidity, polarity, and fragment-level fingerprints, further contributed to the model output. This confirms that fluorescence lifetime is not governed by a single molecular property, but by a coupled interplay between electronic structure, molecular shape, solvent environment, and excited-state relaxation pathways.

From a methodological perspective, the use of cluster-stratified cross-validation and Optuna-based hyperparameter optimization improved model robustness across chemically diverse regions of the dataset. Benchmarking against alternative regression algorithms further confirmed the nonlinear nature of fluorescence lifetime prediction, with ensemble-based methods outperforming conventional linear models. LightGBM was therefore selected as a suitable compromise between predictive accuracy, computational efficiency, scalability, and interpretability.

The error analysis showed that model reliability depends strongly on the underlying lifetime distribution. Predictions were most stable in the intermediate lifetime region, where data density was highest and descriptor–target relationships were better represented. Larger deviations occurred in the sub-nanosecond regime, where relative error is amplified by very small lifetime values, and in the long-lifetime regime, where fewer representative training examples are available. These limitations define the current applicability domain of the model rather than indicating a general failure of the approach.

External validation against TD-DFT and independent experimental data further supports this interpretation. TD-DFT provides a physics-based description of excited states and radiative transitions, but remains computationally demanding and sensitive to methodological choices such as functional, basis set, solvent model, and treatment of vibronic and non-radiative effects. In contrast, the ML model provides rapid lifetime estimates directly from molecular and photophysical descriptors. Although it does not fully replace detailed TD-DFT calculations or experimental spectroscopy, it can serve as a practical surrogate for screening, ranking, and prioritizing candidate fluorophores.

Overall, the main strength of the model lies in its ability to capture global structure–property trends across broad chemical space. This makes it particularly useful for large-scale fluorescence analysis, where the objective is to identify promising compounds and guide experimental or theoretical follow-up rather than achieve exact pointwise agreement for every molecule. The model therefore offers a low-cost and interpretable tool for accelerating fluorophore discovery in sensing, imaging, optoelectronics, and related photophysical applications.

Future improvements should focus on integrating richer experimental metadata, including pH, temperature, ionic strength, oxygen concentration, viscosity, and more detailed solvent descriptors. Expanding underrepresented regions of chemical space, especially very short- and long-lifetime chromophores, would further improve generalization. A particularly promising direction is the development of a hybrid photophysical database combining experimental measurements with standardized TD-DFT-derived descriptors, such as excitation energies, oscillator strengths, HOMO–LUMO gaps, and charge-transfer indicators. Such a database would enable more physically informed machine learning models and could support future multi-task or physics-guided approaches for photophysical property prediction.

## Supplementary Information

Below is the link to the electronic supplementary material.

Supplementary Material 1 (DOCX 78.3 KB)

## Data Availability

Relevant data has been included in the manuscript and supporting information file.The source code associated with this work is available at the GitHub repository: https://github.com/DragosVovea/FluorescenceLifetimePredictor . The repository contains the prediction script, documentation, and example input format required to run the fluorescence lifetime prediction workflow. The trained model files required to reproduce the predictions, including FinalModel.pkl and FinalKMeans.pkl and the python code are deposited on Zenodo and are available at: 10.5281/zenodo.20208152.
