# Supplementary material for: Tree-Based Machine Learning Model for Fluorescence Lifetime Prediction in Organic Compounds
Source: J Fluoresc. 2026 Jun 19;36(6):4121–39. doi: 10.1007/s10895-026-04839-9 (PMC13331902; doi:10.1007/s10895-026-04839-9)
Supplement: Supplementary file 1 — Supplementary Material 1 (DOCX 78.3 KB) [file 10895_2026_4839_MOESM1_ESM.docx]

**Supplementary Information**

Tree-Based Machine Learning Model for Fluorescence Lifetime Prediction in Organic Compounds

Dragoș-Cătălin Vovea^1^, Vasile Chiș^1*^

^1^Faculty of Physics, Babeș-Bolyai University, Str. M. Kogălniceanu 1, RO-400084 Cluj-Napoca, Romania

*Corresponding author, email: [vasile.chis@ubbcluj.ro](mailto:vasile.chis@ubbcluj.ro)

GitHub repository: <https://github.com/DragosVovea/FluorescenceLifetimePredictor>

Zenodo repository:  [10.5281/zenodo.20208151](https://doi.org/10.5281/zenodo.20208151)

This supplementary file provides the workflow scheme, model benchmarking results, optimized LightGBM hyperparameters, cross-validation fold metrics, the 639 descriptors retained after recursive feature elimination, and the external experimental validation references used in the manuscript.

# Model benchmarking

Multiple regression algorithms were benchmarked using the same preprocessing workflow. The LazyPredict results are provided in Table S1. The ranking is sorted by R² score. Because the descriptor space is high-dimensional, adjusted R² values can be unstable and are reported only for completeness; final model selection prioritized R², RMSE, computational efficiency, scalability, and interpretability.

Table S1. LazyPredict benchmarking results for the tested regression algorithms.

| **Model** | **Adjusted R²** | **R²** | **RMSE** | **Time (s)** |
| --- | --- | --- | --- | --- |
| ExtraTreesRegressor | 1.0922 | 0.7678 | 0.8282 | 61.052 |
| HistGradientBoostingRegressor | 1.1051 | 0.7352 | 0.8845 | 6.705 |
| LGBMRegressor | 1.1060 | 0.7329 | 0.8883 | 1.233 |
| RandomForestRegressor | 1.1093 | 0.7245 | 0.9021 | 55.484 |
| BaggingRegressor | 1.1253 | 0.6844 | 0.9655 | 6.100 |
| MLPRegressor | 1.1480 | 0.6271 | 1.0495 | 23.589 |
| KNeighborsRegressor | 1.1589 | 0.5995 | 1.0876 | 0.444 |
| GradientBoostingRegressor | 1.1676 | 0.5777 | 1.1169 | 20.427 |
| LassoCV | 1.1744 | 0.5607 | 1.1392 | 73.213 |
| ElasticNetCV | 1.1754 | 0.5581 | 1.1425 | 41.527 |
| BayesianRidge | 1.1776 | 0.5526 | 1.1496 | 2.476 |
| SVR | 1.1818 | 0.5419 | 1.1633 | 13.985 |
| NuSVR | 1.1835 | 0.5377 | 1.1685 | 15.339 |
| RidgeCV | 1.1914 | 0.5177 | 1.1936 | 2.486 |
| PoissonRegressor | 1.1954 | 0.5077 | 1.2059 | 0.408 |
| OrthogonalMatchingPursuit | 1.1962 | 0.5058 | 1.2082 | 0.377 |
| OrthogonalMatchingPursuitCV | 1.1962 | 0.5058 | 1.2082 | 2.341 |
| Ridge | 1.2003 | 0.4953 | 1.2210 | 0.395 |
| ExtraTreeRegressor | 1.2042 | 0.4856 | 1.2326 | 0.810 |
| HuberRegressor | 1.2150 | 0.4582 | 1.2651 | 5.661 |
| TweedieRegressor | 1.2197 | 0.4463 | 1.2788 | 0.346 |
| GammaRegressor | 1.2244 | 0.4346 | 1.2923 | 1.726 |
| LassoLarsIC | 1.2275 | 0.4268 | 1.3012 | 2.512 |
| LassoLarsCV | 1.2275 | 0.4268 | 1.3012 | 3.742 |
| LarsCV | 1.2285 | 0.4242 | 1.3041 | 4.660 |
| DecisionTreeRegressor | 1.2550 | 0.3576 | 1.3776 | 1.079 |
| LinearSVR | 1.2791 | 0.2969 | 1.4411 | 14.711 |
| AdaBoostRegressor | 1.3161 | 0.2036 | 1.5338 | 16.211 |
| PassiveAggressiveRegressor | 1.3186 | 0.1973 | 1.5398 | 0.415 |
| ElasticNet | 1.3843 | 0.0319 | 1.6910 | 0.248 |
| DummyRegressor | 1.3969 | -5.028e-05 | 1.7187 | 0.209 |
| Lasso | 1.3969 | -5.028e-05 | 1.7187 | 0.298 |
| LassoLars | 1.3969 | -5.028e-05 | 1.7187 | 0.490 |
| QuantileRegressor | 1.4132 | -0.0410 | 1.7535 | 9.221 |
| KernelRidge | 1.8673 | -1.1851 | 2.5405 | 1.214 |
| GaussianProcessRegressor | 2.1161 | -1.8121 | 2.8821 | 116.660 |
| LinearRegression | 4.491e+06 | -1.132e+07 | 5781.3775 | 2.358 |
| TransformedTargetRegressor | 4.491e+06 | -1.132e+07 | 5781.3775 | 1.388 |
| RANSACRegressor | 8.869e+06 | -2.235e+07 | 8124.3210 | 83.166 |
| SGDRegressor | 1.215e+22 | -3.061e+22 | 3.007e+11 | 0.755 |
| Lars | 1.108e+40 | -2.791e+40 | 2.871e+20 | 0.995 |

# Optimized LightGBM hyperparameters

The final LightGBM model was optimized using Optuna. The optimized hyperparameters used for model training are summarized in Table S2.

Table S2. Optimized LightGBM hyperparameters obtained using Optuna.

| **Parameter** | **Value** |
| --- | --- |
| learning_rate | 0.0057 |
| num_leaves | 58 |
| max_depth | 9 |
| min_child_samples | 20 |
| lambda_l1 | 2.535e-05 |
| lambda_l2 | 8.623e-05 |
| feature_fraction | 0.826 |
| bagging_fraction | 0.871 |
| bagging_freq | 2 |
| n_estimators | 16000 |

# Cross-validation fold metrics

Model performance was evaluated using cluster-stratified 10-fold cross-validation. The fold-wise metrics are shown in Table S3. MAE is reported in nanoseconds, while R² is dimensionless.

Table S3. Fold-wise cross-validation performance of the optimized LightGBM model.

| **Fold** | **MAE** | **R²** | **Mean MAE** | **Mean R²** | **MAE dev.** | **R² dev.** | **MAE dev. (%)** | **R² dev. (%)** |
| --- | --- | --- | --- | --- | --- | --- | --- | --- |
| 1 | 0.8257 | 0.7838 | 0.8324 | 0.7523 | -0.0067 | 0.0315 | -0.81 | 4.18 |
| 2 | 0.8207 | 0.7253 | 0.8324 | 0.7523 | -0.0117 | -0.0270 | -1.41 | -3.59 |
| 3 | 0.8474 | 0.7554 | 0.8324 | 0.7523 | 0.0149 | 0.0031 | 1.79 | 0.41 |
| 4 | 0.7533 | 0.7549 | 0.8324 | 0.7523 | -0.0792 | 0.0027 | -9.51 | 0.35 |
| 5 | 0.7120 | 0.8063 | 0.8324 | 0.7523 | -0.1204 | 0.0540 | -14.47 | 7.18 |
| 6 | 0.9092 | 0.7238 | 0.8324 | 0.7523 | 0.0767 | -0.0285 | 9.22 | -3.79 |
| 7 | 0.9218 | 0.7117 | 0.8324 | 0.7523 | 0.0894 | -0.0406 | 10.74 | -5.39 |
| 8 | 0.8192 | 0.7973 | 0.8324 | 0.7523 | -0.0132 | 0.0450 | -1.59 | 5.98 |
| 9 | 0.8296 | 0.7391 | 0.8324 | 0.7523 | -0.0029 | -0.0132 | -0.35 | -1.75 |
| 10 | 0.8855 | 0.7254 | 0.8324 | 0.7523 | 0.0531 | -0.0269 | 6.38 | -3.57 |

# Recursive feature elimination and selected descriptor set

Recursive feature elimination (RFE) was used to reduce descriptor dimensionality while retaining predictive information. The retained 639-feature subset corresponded to iteration 5 of the RFE procedure, with an MAE of 0.8358 ns in the RFE evaluation. The selected descriptors include original photophysical variables, RDKit chromophore and solvent descriptors, molecular fingerprints, 3D geometrical terms, predicted pKa, and engineered interaction descriptors.

Table S4. RFE performance across successive descriptor subsets.

| **RFE iteration** | **Number of features** | **MAE (ns)** |
| --- | --- | --- |
| 1 | 1439 | 0.8392 |
| 2 | 1239 | 0.8373 |
| 3 | 1039 | 0.8370 |
| 4 | 839 | 0.8388 |
| 5 | 639 | 0.8358 |
| 6 | 439 | 0.8412 |

Table S5. Descriptor-group composition of the 639-feature RFE-selected subset.

| **Descriptor group** | **Number of retained features** |
| --- | --- |
| Morgan fingerprint | 268 |
| Chromophore RDKit descriptor | 154 |
| MACCS fingerprint | 90 |
| Solvent RDKit descriptor | 82 |
| Engineered / interaction descriptor | 21 |
| Solvent 3D geometry | 10 |
| Chromophore 3D geometry | 9 |
| Original photophysical / dataset variable | 4 |
| Predicted pKa | 1 |

Table S6. Complete list of the 639 features retained after recursive feature elimination.

| **No.** | **Selected feature** | **Descriptor group** |
| --- | --- | --- |
| 1 | Absorption max (nm) | Original photophysical / dataset variable |
| 2 | Emission max (nm) | Original photophysical / dataset variable |
| 3 | Quantum yield | Original photophysical / dataset variable |
| 4 | Molecular weight (g mol-1) | Original photophysical / dataset variable |
| 5 | Predicted_pKa | Predicted pKa |
| 6 | Chrom_MaxAbsEStateIndex | Chromophore RDKit descriptor |
| 7 | Chrom_MaxEStateIndex | Chromophore RDKit descriptor |
| 8 | Chrom_MinAbsEStateIndex | Chromophore RDKit descriptor |
| 9 | Chrom_MinEStateIndex | Chromophore RDKit descriptor |
| 10 | Chrom_qed | Chromophore RDKit descriptor |
| 11 | Chrom_SPS | Chromophore RDKit descriptor |
| 12 | Chrom_MolWt | Chromophore RDKit descriptor |
| 13 | Chrom_HeavyAtomMolWt | Chromophore RDKit descriptor |
| 14 | Chrom_ExactMolWt | Chromophore RDKit descriptor |
| 15 | Chrom_NumValenceElectrons | Chromophore RDKit descriptor |
| 16 | Chrom_MaxPartialCharge | Chromophore RDKit descriptor |
| 17 | Chrom_MinPartialCharge | Chromophore RDKit descriptor |
| 18 | Chrom_MaxAbsPartialCharge | Chromophore RDKit descriptor |
| 19 | Chrom_MinAbsPartialCharge | Chromophore RDKit descriptor |
| 20 | Chrom_FpDensityMorgan1 | Morgan fingerprint |
| 21 | Chrom_FpDensityMorgan2 | Morgan fingerprint |
| 22 | Chrom_FpDensityMorgan3 | Morgan fingerprint |
| 23 | Chrom_BCUT2D_MWHI | Chromophore RDKit descriptor |
| 24 | Chrom_BCUT2D_MWLOW | Chromophore RDKit descriptor |
| 25 | Chrom_BCUT2D_CHGHI | Chromophore RDKit descriptor |
| 26 | Chrom_BCUT2D_CHGLO | Chromophore RDKit descriptor |
| 27 | Chrom_BCUT2D_LOGPHI | Chromophore RDKit descriptor |
| 28 | Chrom_BCUT2D_LOGPLOW | Chromophore RDKit descriptor |
| 29 | Chrom_BCUT2D_MRHI | Chromophore RDKit descriptor |
| 30 | Chrom_BCUT2D_MRLOW | Chromophore RDKit descriptor |
| 31 | Chrom_AvgIpc | Chromophore RDKit descriptor |
| 32 | Chrom_BalabanJ | Chromophore RDKit descriptor |
| 33 | Chrom_BertzCT | Chromophore RDKit descriptor |
| 34 | Chrom_Chi0 | Chromophore RDKit descriptor |
| 35 | Chrom_Chi0n | Chromophore RDKit descriptor |
| 36 | Chrom_Chi0v | Chromophore RDKit descriptor |
| 37 | Chrom_Chi1 | Chromophore RDKit descriptor |
| 38 | Chrom_Chi1n | Chromophore RDKit descriptor |
| 39 | Chrom_Chi1v | Chromophore RDKit descriptor |
| 40 | Chrom_Chi2n | Chromophore RDKit descriptor |
| 41 | Chrom_Chi2v | Chromophore RDKit descriptor |
| 42 | Chrom_Chi3n | Chromophore RDKit descriptor |
| 43 | Chrom_Chi3v | Chromophore RDKit descriptor |
| 44 | Chrom_Chi4n | Chromophore RDKit descriptor |
| 45 | Chrom_Chi4v | Chromophore RDKit descriptor |
| 46 | Chrom_HallKierAlpha | Chromophore RDKit descriptor |
| 47 | Chrom_Ipc | Chromophore RDKit descriptor |
| 48 | Chrom_Kappa1 | Chromophore RDKit descriptor |
| 49 | Chrom_Kappa2 | Chromophore RDKit descriptor |
| 50 | Chrom_Kappa3 | Chromophore RDKit descriptor |
| 51 | Chrom_LabuteASA | Chromophore RDKit descriptor |
| 52 | Chrom_PEOE_VSA1 | Chromophore RDKit descriptor |
| 53 | Chrom_PEOE_VSA10 | Chromophore RDKit descriptor |
| 54 | Chrom_PEOE_VSA11 | Chromophore RDKit descriptor |
| 55 | Chrom_PEOE_VSA12 | Chromophore RDKit descriptor |
| 56 | Chrom_PEOE_VSA13 | Chromophore RDKit descriptor |
| 57 | Chrom_PEOE_VSA14 | Chromophore RDKit descriptor |
| 58 | Chrom_PEOE_VSA2 | Chromophore RDKit descriptor |
| 59 | Chrom_PEOE_VSA3 | Chromophore RDKit descriptor |
| 60 | Chrom_PEOE_VSA4 | Chromophore RDKit descriptor |
| 61 | Chrom_PEOE_VSA5 | Chromophore RDKit descriptor |
| 62 | Chrom_PEOE_VSA6 | Chromophore RDKit descriptor |
| 63 | Chrom_PEOE_VSA7 | Chromophore RDKit descriptor |
| 64 | Chrom_PEOE_VSA8 | Chromophore RDKit descriptor |
| 65 | Chrom_PEOE_VSA9 | Chromophore RDKit descriptor |
| 66 | Chrom_SMR_VSA1 | Chromophore RDKit descriptor |
| 67 | Chrom_SMR_VSA10 | Chromophore RDKit descriptor |
| 68 | Chrom_SMR_VSA2 | Chromophore RDKit descriptor |
| 69 | Chrom_SMR_VSA3 | Chromophore RDKit descriptor |
| 70 | Chrom_SMR_VSA4 | Chromophore RDKit descriptor |
| 71 | Chrom_SMR_VSA5 | Chromophore RDKit descriptor |
| 72 | Chrom_SMR_VSA6 | Chromophore RDKit descriptor |
| 73 | Chrom_SMR_VSA7 | Chromophore RDKit descriptor |
| 74 | Chrom_SMR_VSA9 | Chromophore RDKit descriptor |
| 75 | Chrom_SlogP_VSA1 | Chromophore RDKit descriptor |
| 76 | Chrom_SlogP_VSA10 | Chromophore RDKit descriptor |
| 77 | Chrom_SlogP_VSA11 | Chromophore RDKit descriptor |
| 78 | Chrom_SlogP_VSA12 | Chromophore RDKit descriptor |
| 79 | Chrom_SlogP_VSA2 | Chromophore RDKit descriptor |
| 80 | Chrom_SlogP_VSA3 | Chromophore RDKit descriptor |
| 81 | Chrom_SlogP_VSA4 | Chromophore RDKit descriptor |
| 82 | Chrom_SlogP_VSA5 | Chromophore RDKit descriptor |
| 83 | Chrom_SlogP_VSA6 | Chromophore RDKit descriptor |
| 84 | Chrom_SlogP_VSA8 | Chromophore RDKit descriptor |
| 85 | Chrom_TPSA | Chromophore RDKit descriptor |
| 86 | Chrom_EState_VSA1 | Chromophore RDKit descriptor |
| 87 | Chrom_EState_VSA10 | Chromophore RDKit descriptor |
| 88 | Chrom_EState_VSA2 | Chromophore RDKit descriptor |
| 89 | Chrom_EState_VSA3 | Chromophore RDKit descriptor |
| 90 | Chrom_EState_VSA4 | Chromophore RDKit descriptor |
| 91 | Chrom_EState_VSA5 | Chromophore RDKit descriptor |
| 92 | Chrom_EState_VSA6 | Chromophore RDKit descriptor |
| 93 | Chrom_EState_VSA7 | Chromophore RDKit descriptor |
| 94 | Chrom_EState_VSA8 | Chromophore RDKit descriptor |
| 95 | Chrom_EState_VSA9 | Chromophore RDKit descriptor |
| 96 | Chrom_VSA_EState1 | Chromophore RDKit descriptor |
| 97 | Chrom_VSA_EState10 | Chromophore RDKit descriptor |
| 98 | Chrom_VSA_EState2 | Chromophore RDKit descriptor |
| 99 | Chrom_VSA_EState3 | Chromophore RDKit descriptor |
| 100 | Chrom_VSA_EState4 | Chromophore RDKit descriptor |
| 101 | Chrom_VSA_EState5 | Chromophore RDKit descriptor |
| 102 | Chrom_VSA_EState6 | Chromophore RDKit descriptor |
| 103 | Chrom_VSA_EState7 | Chromophore RDKit descriptor |
| 104 | Chrom_VSA_EState8 | Chromophore RDKit descriptor |
| 105 | Chrom_VSA_EState9 | Chromophore RDKit descriptor |
| 106 | Chrom_FractionCSP3 | Chromophore RDKit descriptor |
| 107 | Chrom_HeavyAtomCount | Chromophore RDKit descriptor |
| 108 | Chrom_NHOHCount | Chromophore RDKit descriptor |
| 109 | Chrom_NOCount | Chromophore RDKit descriptor |
| 110 | Chrom_NumAliphaticCarbocycles | Chromophore RDKit descriptor |
| 111 | Chrom_NumAliphaticHeterocycles | Chromophore RDKit descriptor |
| 112 | Chrom_NumAliphaticRings | Chromophore RDKit descriptor |
| 113 | Chrom_NumAmideBonds | Chromophore RDKit descriptor |
| 114 | Chrom_NumAromaticCarbocycles | Chromophore RDKit descriptor |
| 115 | Chrom_NumAromaticHeterocycles | Chromophore RDKit descriptor |
| 116 | Chrom_NumAromaticRings | Chromophore RDKit descriptor |
| 117 | Chrom_NumAtomStereoCenters | Chromophore RDKit descriptor |
| 118 | Chrom_NumBridgeheadAtoms | Chromophore RDKit descriptor |
| 119 | Chrom_NumHAcceptors | Chromophore RDKit descriptor |
| 120 | Chrom_NumHDonors | Chromophore RDKit descriptor |
| 121 | Chrom_NumHeteroatoms | Chromophore RDKit descriptor |
| 122 | Chrom_NumHeterocycles | Chromophore RDKit descriptor |
| 123 | Chrom_NumRotatableBonds | Chromophore RDKit descriptor |
| 124 | Chrom_NumSaturatedHeterocycles | Chromophore RDKit descriptor |
| 125 | Chrom_NumSaturatedRings | Chromophore RDKit descriptor |
| 126 | Chrom_NumUnspecifiedAtomStereoCenters | Chromophore RDKit descriptor |
| 127 | Chrom_Phi | Chromophore RDKit descriptor |
| 128 | Chrom_RingCount | Chromophore RDKit descriptor |
| 129 | Chrom_MolLogP | Chromophore RDKit descriptor |
| 130 | Chrom_MolMR | Chromophore RDKit descriptor |
| 131 | Chrom_fr_ArN | Chromophore RDKit descriptor |
| 132 | Chrom_fr_Ar_N | Chromophore RDKit descriptor |
| 133 | Chrom_fr_Ar_NH | Chromophore RDKit descriptor |
| 134 | Chrom_fr_COO | Chromophore RDKit descriptor |
| 135 | Chrom_fr_C_O | Chromophore RDKit descriptor |
| 136 | Chrom_fr_C_O_noCOO | Chromophore RDKit descriptor |
| 137 | Chrom_fr_Imine | Chromophore RDKit descriptor |
| 138 | Chrom_fr_NH0 | Chromophore RDKit descriptor |
| 139 | Chrom_fr_NH1 | Chromophore RDKit descriptor |
| 140 | Chrom_fr_Ndealkylation1 | Chromophore RDKit descriptor |
| 141 | Chrom_fr_Ndealkylation2 | Chromophore RDKit descriptor |
| 142 | Chrom_fr_Nhpyrrole | Chromophore RDKit descriptor |
| 143 | Chrom_fr_allylic_oxid | Chromophore RDKit descriptor |
| 144 | Chrom_fr_aniline | Chromophore RDKit descriptor |
| 145 | Chrom_fr_aryl_methyl | Chromophore RDKit descriptor |
| 146 | Chrom_fr_benzene | Chromophore RDKit descriptor |
| 147 | Chrom_fr_bicyclic | Chromophore RDKit descriptor |
| 148 | Chrom_fr_ester | Chromophore RDKit descriptor |
| 149 | Chrom_fr_ether | Chromophore RDKit descriptor |
| 150 | Chrom_fr_halogen | Chromophore RDKit descriptor |
| 151 | Chrom_fr_imidazole | Chromophore RDKit descriptor |
| 152 | Chrom_fr_imide | Chromophore RDKit descriptor |
| 153 | Chrom_fr_ketone | Chromophore RDKit descriptor |
| 154 | Chrom_fr_methoxy | Chromophore RDKit descriptor |
| 155 | Chrom_fr_nitrile | Chromophore RDKit descriptor |
| 156 | Chrom_fr_nitro_arom_nonortho | Chromophore RDKit descriptor |
| 157 | Chrom_fr_para_hydroxylation | Chromophore RDKit descriptor |
| 158 | Chrom_fr_pyridine | Chromophore RDKit descriptor |
| 159 | Chrom_fr_thiophene | Chromophore RDKit descriptor |
| 160 | Chrom_fr_unbrch_alkane | Chromophore RDKit descriptor |
| 161 | Chrom_MaxCharge | Chromophore RDKit descriptor |
| 162 | Chrom_MinCharge | Chromophore RDKit descriptor |
| 163 | Solv_MaxAbsEStateIndex | Solvent RDKit descriptor |
| 164 | Solv_MaxEStateIndex | Solvent RDKit descriptor |
| 165 | Solv_MinAbsEStateIndex | Solvent RDKit descriptor |
| 166 | Solv_MinEStateIndex | Solvent RDKit descriptor |
| 167 | Solv_qed | Solvent RDKit descriptor |
| 168 | Solv_SPS | Solvent RDKit descriptor |
| 169 | Solv_HeavyAtomMolWt | Solvent RDKit descriptor |
| 170 | Solv_ExactMolWt | Solvent RDKit descriptor |
| 171 | Solv_NumValenceElectrons | Solvent RDKit descriptor |
| 172 | Solv_MaxPartialCharge | Solvent RDKit descriptor |
| 173 | Solv_MinPartialCharge | Solvent RDKit descriptor |
| 174 | Solv_MaxAbsPartialCharge | Solvent RDKit descriptor |
| 175 | Solv_MinAbsPartialCharge | Solvent RDKit descriptor |
| 176 | Solv_FpDensityMorgan1 | Morgan fingerprint |
| 177 | Solv_FpDensityMorgan2 | Morgan fingerprint |
| 178 | Solv_FpDensityMorgan3 | Morgan fingerprint |
| 179 | Solv_BCUT2D_MWHI | Solvent RDKit descriptor |
| 180 | Solv_BCUT2D_MWLOW | Solvent RDKit descriptor |
| 181 | Solv_BCUT2D_CHGHI | Solvent RDKit descriptor |
| 182 | Solv_BCUT2D_CHGLO | Solvent RDKit descriptor |
| 183 | Solv_BCUT2D_LOGPHI | Solvent RDKit descriptor |
| 184 | Solv_BCUT2D_LOGPLOW | Solvent RDKit descriptor |
| 185 | Solv_BCUT2D_MRHI | Solvent RDKit descriptor |
| 186 | Solv_BCUT2D_MRLOW | Solvent RDKit descriptor |
| 187 | Solv_AvgIpc | Solvent RDKit descriptor |
| 188 | Solv_BalabanJ | Solvent RDKit descriptor |
| 189 | Solv_BertzCT | Solvent RDKit descriptor |
| 190 | Solv_Chi0n | Solvent RDKit descriptor |
| 191 | Solv_Chi0v | Solvent RDKit descriptor |
| 192 | Solv_Chi1n | Solvent RDKit descriptor |
| 193 | Solv_Chi1v | Solvent RDKit descriptor |
| 194 | Solv_Chi2n | Solvent RDKit descriptor |
| 195 | Solv_Chi2v | Solvent RDKit descriptor |
| 196 | Solv_Chi3n | Solvent RDKit descriptor |
| 197 | Solv_Chi3v | Solvent RDKit descriptor |
| 198 | Solv_HallKierAlpha | Solvent RDKit descriptor |
| 199 | Solv_Ipc | Solvent RDKit descriptor |
| 200 | Solv_Kappa2 | Solvent RDKit descriptor |
| 201 | Solv_LabuteASA | Solvent RDKit descriptor |
| 202 | Solv_PEOE_VSA1 | Solvent RDKit descriptor |
| 203 | Solv_PEOE_VSA11 | Solvent RDKit descriptor |
| 204 | Solv_PEOE_VSA5 | Solvent RDKit descriptor |
| 205 | Solv_PEOE_VSA6 | Solvent RDKit descriptor |
| 206 | Solv_PEOE_VSA7 | Solvent RDKit descriptor |
| 207 | Solv_PEOE_VSA8 | Solvent RDKit descriptor |
| 208 | Solv_PEOE_VSA9 | Solvent RDKit descriptor |
| 209 | Solv_SMR_VSA1 | Solvent RDKit descriptor |
| 210 | Solv_SMR_VSA10 | Solvent RDKit descriptor |
| 211 | Solv_SMR_VSA5 | Solvent RDKit descriptor |
| 212 | Solv_SMR_VSA6 | Solvent RDKit descriptor |
| 213 | Solv_SlogP_VSA1 | Solvent RDKit descriptor |
| 214 | Solv_SlogP_VSA12 | Solvent RDKit descriptor |
| 215 | Solv_SlogP_VSA2 | Solvent RDKit descriptor |
| 216 | Solv_SlogP_VSA3 | Solvent RDKit descriptor |
| 217 | Solv_SlogP_VSA5 | Solvent RDKit descriptor |
| 218 | Solv_SlogP_VSA8 | Solvent RDKit descriptor |
| 219 | Solv_EState_VSA1 | Solvent RDKit descriptor |
| 220 | Solv_EState_VSA10 | Solvent RDKit descriptor |
| 221 | Solv_EState_VSA2 | Solvent RDKit descriptor |
| 222 | Solv_EState_VSA3 | Solvent RDKit descriptor |
| 223 | Solv_EState_VSA4 | Solvent RDKit descriptor |
| 224 | Solv_EState_VSA5 | Solvent RDKit descriptor |
| 225 | Solv_EState_VSA6 | Solvent RDKit descriptor |
| 226 | Solv_EState_VSA7 | Solvent RDKit descriptor |
| 227 | Solv_EState_VSA8 | Solvent RDKit descriptor |
| 228 | Solv_EState_VSA9 | Solvent RDKit descriptor |
| 229 | Solv_VSA_EState1 | Solvent RDKit descriptor |
| 230 | Solv_VSA_EState2 | Solvent RDKit descriptor |
| 231 | Solv_VSA_EState3 | Solvent RDKit descriptor |
| 232 | Solv_VSA_EState4 | Solvent RDKit descriptor |
| 233 | Solv_VSA_EState6 | Solvent RDKit descriptor |
| 234 | Solv_VSA_EState7 | Solvent RDKit descriptor |
| 235 | Solv_VSA_EState8 | Solvent RDKit descriptor |
| 236 | Solv_VSA_EState9 | Solvent RDKit descriptor |
| 237 | Solv_NHOHCount | Solvent RDKit descriptor |
| 238 | Solv_NumHAcceptors | Solvent RDKit descriptor |
| 239 | Solv_NumHeteroatoms | Solvent RDKit descriptor |
| 240 | Solv_Phi | Solvent RDKit descriptor |
| 241 | Solv_MolMR | Solvent RDKit descriptor |
| 242 | Solv_fr_alkyl_halide | Solvent RDKit descriptor |
| 243 | Solv_fr_ether | Solvent RDKit descriptor |
| 244 | Solv_fr_halogen | Solvent RDKit descriptor |
| 245 | Solv_MaxCharge | Solvent RDKit descriptor |
| 246 | Solv_MinCharge | Solvent RDKit descriptor |
| 247 | Solv_MeanCharge | Solvent RDKit descriptor |
| 248 | RigidityScore | Engineered / interaction descriptor |
| 249 | HbondPotential | Engineered / interaction descriptor |
| 250 | PolarityRatio | Engineered / interaction descriptor |
| 251 | AromaticAliphaticRatio | Engineered / interaction descriptor |
| 252 | PolarityDifference | Engineered / interaction descriptor |
| 253 | C3_BL_Max | Chromophore 3D geometry |
| 254 | C3_BL_Min | Chromophore 3D geometry |
| 255 | C3_Ang_Mean | Chromophore 3D geometry |
| 256 | C3_Ang_Max | Chromophore 3D geometry |
| 257 | C3_Ang_Min | Chromophore 3D geometry |
| 258 | C3_Dih_Mean | Chromophore 3D geometry |
| 259 | C3_Dih_Std | Chromophore 3D geometry |
| 260 | C3_Plan_RMSD | Chromophore 3D geometry |
| 261 | C3_Rg | Chromophore 3D geometry |
| 262 | S3_BL_Mean | Solvent 3D geometry |
| 263 | S3_BL_Max | Solvent 3D geometry |
| 264 | S3_BL_Min | Solvent 3D geometry |
| 265 | S3_Ang_Mean | Solvent 3D geometry |
| 266 | S3_Ang_Max | Solvent 3D geometry |
| 267 | S3_Ang_Min | Solvent 3D geometry |
| 268 | S3_Dih_Mean | Solvent 3D geometry |
| 269 | S3_Dih_Std | Solvent 3D geometry |
| 270 | S3_Plan_RMSD | Solvent 3D geometry |
| 271 | S3_Rg | Solvent 3D geometry |
| 272 | Chrom_Morgan_0 | Morgan fingerprint |
| 273 | Chrom_Morgan_1 | Morgan fingerprint |
| 274 | Chrom_Morgan_2 | Morgan fingerprint |
| 275 | Chrom_Morgan_4 | Morgan fingerprint |
| 276 | Chrom_Morgan_7 | Morgan fingerprint |
| 277 | Chrom_Morgan_9 | Morgan fingerprint |
| 278 | Chrom_Morgan_13 | Morgan fingerprint |
| 279 | Chrom_Morgan_14 | Morgan fingerprint |
| 280 | Chrom_Morgan_15 | Morgan fingerprint |
| 281 | Chrom_Morgan_23 | Morgan fingerprint |
| 282 | Chrom_Morgan_25 | Morgan fingerprint |
| 283 | Chrom_Morgan_31 | Morgan fingerprint |
| 284 | Chrom_Morgan_33 | Morgan fingerprint |
| 285 | Chrom_Morgan_34 | Morgan fingerprint |
| 286 | Chrom_Morgan_35 | Morgan fingerprint |
| 287 | Chrom_Morgan_36 | Morgan fingerprint |
| 288 | Chrom_Morgan_37 | Morgan fingerprint |
| 289 | Chrom_Morgan_43 | Morgan fingerprint |
| 290 | Chrom_Morgan_44 | Morgan fingerprint |
| 291 | Chrom_Morgan_45 | Morgan fingerprint |
| 292 | Chrom_Morgan_47 | Morgan fingerprint |
| 293 | Chrom_Morgan_49 | Morgan fingerprint |
| 294 | Chrom_Morgan_52 | Morgan fingerprint |
| 295 | Chrom_Morgan_54 | Morgan fingerprint |
| 296 | Chrom_Morgan_60 | Morgan fingerprint |
| 297 | Chrom_Morgan_62 | Morgan fingerprint |
| 298 | Chrom_Morgan_63 | Morgan fingerprint |
| 299 | Chrom_Morgan_64 | Morgan fingerprint |
| 300 | Chrom_Morgan_66 | Morgan fingerprint |
| 301 | Chrom_Morgan_68 | Morgan fingerprint |
| 302 | Chrom_Morgan_71 | Morgan fingerprint |
| 303 | Chrom_Morgan_72 | Morgan fingerprint |
| 304 | Chrom_Morgan_73 | Morgan fingerprint |
| 305 | Chrom_Morgan_74 | Morgan fingerprint |
| 306 | Chrom_Morgan_75 | Morgan fingerprint |
| 307 | Chrom_Morgan_76 | Morgan fingerprint |
| 308 | Chrom_Morgan_80 | Morgan fingerprint |
| 309 | Chrom_Morgan_82 | Morgan fingerprint |
| 310 | Chrom_Morgan_83 | Morgan fingerprint |
| 311 | Chrom_Morgan_84 | Morgan fingerprint |
| 312 | Chrom_Morgan_86 | Morgan fingerprint |
| 313 | Chrom_Morgan_94 | Morgan fingerprint |
| 314 | Chrom_Morgan_97 | Morgan fingerprint |
| 315 | Chrom_Morgan_102 | Morgan fingerprint |
| 316 | Chrom_Morgan_103 | Morgan fingerprint |
| 317 | Chrom_Morgan_104 | Morgan fingerprint |
| 318 | Chrom_Morgan_105 | Morgan fingerprint |
| 319 | Chrom_Morgan_106 | Morgan fingerprint |
| 320 | Chrom_Morgan_112 | Morgan fingerprint |
| 321 | Chrom_Morgan_113 | Morgan fingerprint |
| 322 | Chrom_Morgan_114 | Morgan fingerprint |
| 323 | Chrom_Morgan_117 | Morgan fingerprint |
| 324 | Chrom_Morgan_118 | Morgan fingerprint |
| 325 | Chrom_Morgan_119 | Morgan fingerprint |
| 326 | Chrom_Morgan_121 | Morgan fingerprint |
| 327 | Chrom_Morgan_122 | Morgan fingerprint |
| 328 | Chrom_Morgan_124 | Morgan fingerprint |
| 329 | Chrom_Morgan_126 | Morgan fingerprint |
| 330 | Chrom_Morgan_128 | Morgan fingerprint |
| 331 | Chrom_Morgan_129 | Morgan fingerprint |
| 332 | Chrom_Morgan_130 | Morgan fingerprint |
| 333 | Chrom_Morgan_131 | Morgan fingerprint |
| 334 | Chrom_Morgan_136 | Morgan fingerprint |
| 335 | Chrom_Morgan_137 | Morgan fingerprint |
| 336 | Chrom_Morgan_138 | Morgan fingerprint |
| 337 | Chrom_Morgan_139 | Morgan fingerprint |
| 338 | Chrom_Morgan_144 | Morgan fingerprint |
| 339 | Chrom_Morgan_145 | Morgan fingerprint |
| 340 | Chrom_Morgan_147 | Morgan fingerprint |
| 341 | Chrom_Morgan_148 | Morgan fingerprint |
| 342 | Chrom_Morgan_150 | Morgan fingerprint |
| 343 | Chrom_Morgan_151 | Morgan fingerprint |
| 344 | Chrom_Morgan_152 | Morgan fingerprint |
| 345 | Chrom_Morgan_155 | Morgan fingerprint |
| 346 | Chrom_Morgan_159 | Morgan fingerprint |
| 347 | Chrom_Morgan_160 | Morgan fingerprint |
| 348 | Chrom_Morgan_161 | Morgan fingerprint |
| 349 | Chrom_Morgan_162 | Morgan fingerprint |
| 350 | Chrom_Morgan_163 | Morgan fingerprint |
| 351 | Chrom_Morgan_164 | Morgan fingerprint |
| 352 | Chrom_Morgan_168 | Morgan fingerprint |
| 353 | Chrom_Morgan_169 | Morgan fingerprint |
| 354 | Chrom_Morgan_172 | Morgan fingerprint |
| 355 | Chrom_Morgan_175 | Morgan fingerprint |
| 356 | Chrom_Morgan_177 | Morgan fingerprint |
| 357 | Chrom_Morgan_179 | Morgan fingerprint |
| 358 | Chrom_Morgan_180 | Morgan fingerprint |
| 359 | Chrom_Morgan_181 | Morgan fingerprint |
| 360 | Chrom_Morgan_182 | Morgan fingerprint |
| 361 | Chrom_Morgan_183 | Morgan fingerprint |
| 362 | Chrom_Morgan_186 | Morgan fingerprint |
| 363 | Chrom_Morgan_187 | Morgan fingerprint |
| 364 | Chrom_Morgan_189 | Morgan fingerprint |
| 365 | Chrom_Morgan_192 | Morgan fingerprint |
| 366 | Chrom_Morgan_194 | Morgan fingerprint |
| 367 | Chrom_Morgan_196 | Morgan fingerprint |
| 368 | Chrom_Morgan_197 | Morgan fingerprint |
| 369 | Chrom_Morgan_199 | Morgan fingerprint |
| 370 | Chrom_Morgan_200 | Morgan fingerprint |
| 371 | Chrom_Morgan_202 | Morgan fingerprint |
| 372 | Chrom_Morgan_203 | Morgan fingerprint |
| 373 | Chrom_Morgan_205 | Morgan fingerprint |
| 374 | Chrom_Morgan_206 | Morgan fingerprint |
| 375 | Chrom_Morgan_209 | Morgan fingerprint |
| 376 | Chrom_Morgan_210 | Morgan fingerprint |
| 377 | Chrom_Morgan_211 | Morgan fingerprint |
| 378 | Chrom_Morgan_213 | Morgan fingerprint |
| 379 | Chrom_Morgan_216 | Morgan fingerprint |
| 380 | Chrom_Morgan_217 | Morgan fingerprint |
| 381 | Chrom_Morgan_219 | Morgan fingerprint |
| 382 | Chrom_Morgan_224 | Morgan fingerprint |
| 383 | Chrom_Morgan_225 | Morgan fingerprint |
| 384 | Chrom_Morgan_226 | Morgan fingerprint |
| 385 | Chrom_Morgan_231 | Morgan fingerprint |
| 386 | Chrom_Morgan_232 | Morgan fingerprint |
| 387 | Chrom_Morgan_233 | Morgan fingerprint |
| 388 | Chrom_Morgan_239 | Morgan fingerprint |
| 389 | Chrom_Morgan_241 | Morgan fingerprint |
| 390 | Chrom_Morgan_243 | Morgan fingerprint |
| 391 | Chrom_Morgan_247 | Morgan fingerprint |
| 392 | Chrom_Morgan_249 | Morgan fingerprint |
| 393 | Chrom_Morgan_250 | Morgan fingerprint |
| 394 | Chrom_Morgan_252 | Morgan fingerprint |
| 395 | Chrom_Morgan_257 | Morgan fingerprint |
| 396 | Chrom_Morgan_261 | Morgan fingerprint |
| 397 | Chrom_Morgan_263 | Morgan fingerprint |
| 398 | Chrom_Morgan_265 | Morgan fingerprint |
| 399 | Chrom_Morgan_267 | Morgan fingerprint |
| 400 | Chrom_Morgan_269 | Morgan fingerprint |
| 401 | Chrom_Morgan_271 | Morgan fingerprint |
| 402 | Chrom_Morgan_272 | Morgan fingerprint |
| 403 | Chrom_Morgan_273 | Morgan fingerprint |
| 404 | Chrom_Morgan_274 | Morgan fingerprint |
| 405 | Chrom_Morgan_276 | Morgan fingerprint |
| 406 | Chrom_Morgan_278 | Morgan fingerprint |
| 407 | Chrom_Morgan_279 | Morgan fingerprint |
| 408 | Chrom_Morgan_280 | Morgan fingerprint |
| 409 | Chrom_Morgan_281 | Morgan fingerprint |
| 410 | Chrom_Morgan_283 | Morgan fingerprint |
| 411 | Chrom_Morgan_286 | Morgan fingerprint |
| 412 | Chrom_Morgan_287 | Morgan fingerprint |
| 413 | Chrom_Morgan_288 | Morgan fingerprint |
| 414 | Chrom_Morgan_289 | Morgan fingerprint |
| 415 | Chrom_Morgan_290 | Morgan fingerprint |
| 416 | Chrom_Morgan_291 | Morgan fingerprint |
| 417 | Chrom_Morgan_292 | Morgan fingerprint |
| 418 | Chrom_Morgan_294 | Morgan fingerprint |
| 419 | Chrom_Morgan_295 | Morgan fingerprint |
| 420 | Chrom_Morgan_298 | Morgan fingerprint |
| 421 | Chrom_Morgan_305 | Morgan fingerprint |
| 422 | Chrom_Morgan_310 | Morgan fingerprint |
| 423 | Chrom_Morgan_314 | Morgan fingerprint |
| 424 | Chrom_Morgan_315 | Morgan fingerprint |
| 425 | Chrom_Morgan_316 | Morgan fingerprint |
| 426 | Chrom_Morgan_318 | Morgan fingerprint |
| 427 | Chrom_Morgan_319 | Morgan fingerprint |
| 428 | Chrom_Morgan_320 | Morgan fingerprint |
| 429 | Chrom_Morgan_321 | Morgan fingerprint |
| 430 | Chrom_Morgan_322 | Morgan fingerprint |
| 431 | Chrom_Morgan_323 | Morgan fingerprint |
| 432 | Chrom_Morgan_325 | Morgan fingerprint |
| 433 | Chrom_Morgan_326 | Morgan fingerprint |
| 434 | Chrom_Morgan_327 | Morgan fingerprint |
| 435 | Chrom_Morgan_329 | Morgan fingerprint |
| 436 | Chrom_Morgan_331 | Morgan fingerprint |
| 437 | Chrom_Morgan_332 | Morgan fingerprint |
| 438 | Chrom_Morgan_333 | Morgan fingerprint |
| 439 | Chrom_Morgan_336 | Morgan fingerprint |
| 440 | Chrom_Morgan_341 | Morgan fingerprint |
| 441 | Chrom_Morgan_342 | Morgan fingerprint |
| 442 | Chrom_Morgan_343 | Morgan fingerprint |
| 443 | Chrom_Morgan_345 | Morgan fingerprint |
| 444 | Chrom_Morgan_352 | Morgan fingerprint |
| 445 | Chrom_Morgan_354 | Morgan fingerprint |
| 446 | Chrom_Morgan_357 | Morgan fingerprint |
| 447 | Chrom_Morgan_358 | Morgan fingerprint |
| 448 | Chrom_Morgan_360 | Morgan fingerprint |
| 449 | Chrom_Morgan_361 | Morgan fingerprint |
| 450 | Chrom_Morgan_362 | Morgan fingerprint |
| 451 | Chrom_Morgan_363 | Morgan fingerprint |
| 452 | Chrom_Morgan_366 | Morgan fingerprint |
| 453 | Chrom_Morgan_367 | Morgan fingerprint |
| 454 | Chrom_Morgan_369 | Morgan fingerprint |
| 455 | Chrom_Morgan_371 | Morgan fingerprint |
| 456 | Chrom_Morgan_376 | Morgan fingerprint |
| 457 | Chrom_Morgan_378 | Morgan fingerprint |
| 458 | Chrom_Morgan_379 | Morgan fingerprint |
| 459 | Chrom_Morgan_381 | Morgan fingerprint |
| 460 | Chrom_Morgan_383 | Morgan fingerprint |
| 461 | Chrom_Morgan_384 | Morgan fingerprint |
| 462 | Chrom_Morgan_385 | Morgan fingerprint |
| 463 | Chrom_Morgan_389 | Morgan fingerprint |
| 464 | Chrom_Morgan_391 | Morgan fingerprint |
| 465 | Chrom_Morgan_392 | Morgan fingerprint |
| 466 | Chrom_Morgan_393 | Morgan fingerprint |
| 467 | Chrom_Morgan_394 | Morgan fingerprint |
| 468 | Chrom_Morgan_396 | Morgan fingerprint |
| 469 | Chrom_Morgan_397 | Morgan fingerprint |
| 470 | Chrom_Morgan_404 | Morgan fingerprint |
| 471 | Chrom_Morgan_405 | Morgan fingerprint |
| 472 | Chrom_Morgan_406 | Morgan fingerprint |
| 473 | Chrom_Morgan_407 | Morgan fingerprint |
| 474 | Chrom_Morgan_410 | Morgan fingerprint |
| 475 | Chrom_Morgan_412 | Morgan fingerprint |
| 476 | Chrom_Morgan_414 | Morgan fingerprint |
| 477 | Chrom_Morgan_415 | Morgan fingerprint |
| 478 | Chrom_Morgan_416 | Morgan fingerprint |
| 479 | Chrom_Morgan_417 | Morgan fingerprint |
| 480 | Chrom_Morgan_418 | Morgan fingerprint |
| 481 | Chrom_Morgan_419 | Morgan fingerprint |
| 482 | Chrom_Morgan_420 | Morgan fingerprint |
| 483 | Chrom_Morgan_421 | Morgan fingerprint |
| 484 | Chrom_Morgan_422 | Morgan fingerprint |
| 485 | Chrom_Morgan_423 | Morgan fingerprint |
| 486 | Chrom_Morgan_424 | Morgan fingerprint |
| 487 | Chrom_Morgan_425 | Morgan fingerprint |
| 488 | Chrom_Morgan_427 | Morgan fingerprint |
| 489 | Chrom_Morgan_428 | Morgan fingerprint |
| 490 | Chrom_Morgan_429 | Morgan fingerprint |
| 491 | Chrom_Morgan_430 | Morgan fingerprint |
| 492 | Chrom_Morgan_432 | Morgan fingerprint |
| 493 | Chrom_Morgan_434 | Morgan fingerprint |
| 494 | Chrom_Morgan_435 | Morgan fingerprint |
| 495 | Chrom_Morgan_436 | Morgan fingerprint |
| 496 | Chrom_Morgan_437 | Morgan fingerprint |
| 497 | Chrom_Morgan_438 | Morgan fingerprint |
| 498 | Chrom_Morgan_439 | Morgan fingerprint |
| 499 | Chrom_Morgan_441 | Morgan fingerprint |
| 500 | Chrom_Morgan_444 | Morgan fingerprint |
| 501 | Chrom_Morgan_446 | Morgan fingerprint |
| 502 | Chrom_Morgan_448 | Morgan fingerprint |
| 503 | Chrom_Morgan_449 | Morgan fingerprint |
| 504 | Chrom_Morgan_452 | Morgan fingerprint |
| 505 | Chrom_Morgan_453 | Morgan fingerprint |
| 506 | Chrom_Morgan_454 | Morgan fingerprint |
| 507 | Chrom_Morgan_455 | Morgan fingerprint |
| 508 | Chrom_Morgan_457 | Morgan fingerprint |
| 509 | Chrom_Morgan_458 | Morgan fingerprint |
| 510 | Chrom_Morgan_462 | Morgan fingerprint |
| 511 | Chrom_Morgan_465 | Morgan fingerprint |
| 512 | Chrom_Morgan_468 | Morgan fingerprint |
| 513 | Chrom_Morgan_471 | Morgan fingerprint |
| 514 | Chrom_Morgan_472 | Morgan fingerprint |
| 515 | Chrom_Morgan_474 | Morgan fingerprint |
| 516 | Chrom_Morgan_475 | Morgan fingerprint |
| 517 | Chrom_Morgan_484 | Morgan fingerprint |
| 518 | Chrom_Morgan_485 | Morgan fingerprint |
| 519 | Chrom_Morgan_489 | Morgan fingerprint |
| 520 | Chrom_Morgan_490 | Morgan fingerprint |
| 521 | Chrom_Morgan_493 | Morgan fingerprint |
| 522 | Chrom_Morgan_497 | Morgan fingerprint |
| 523 | Chrom_Morgan_504 | Morgan fingerprint |
| 524 | Chrom_Morgan_505 | Morgan fingerprint |
| 525 | Chrom_Morgan_507 | Morgan fingerprint |
| 526 | Chrom_Morgan_508 | Morgan fingerprint |
| 527 | Chrom_Morgan_509 | Morgan fingerprint |
| 528 | Chrom_Morgan_511 | Morgan fingerprint |
| 529 | Chrom_MACCS_17 | MACCS fingerprint |
| 530 | Chrom_MACCS_30 | MACCS fingerprint |
| 531 | Chrom_MACCS_36 | MACCS fingerprint |
| 532 | Chrom_MACCS_38 | MACCS fingerprint |
| 533 | Chrom_MACCS_41 | MACCS fingerprint |
| 534 | Chrom_MACCS_42 | MACCS fingerprint |
| 535 | Chrom_MACCS_45 | MACCS fingerprint |
| 536 | Chrom_MACCS_49 | MACCS fingerprint |
| 537 | Chrom_MACCS_50 | MACCS fingerprint |
| 538 | Chrom_MACCS_57 | MACCS fingerprint |
| 539 | Chrom_MACCS_59 | MACCS fingerprint |
| 540 | Chrom_MACCS_62 | MACCS fingerprint |
| 541 | Chrom_MACCS_65 | MACCS fingerprint |
| 542 | Chrom_MACCS_70 | MACCS fingerprint |
| 543 | Chrom_MACCS_72 | MACCS fingerprint |
| 544 | Chrom_MACCS_74 | MACCS fingerprint |
| 545 | Chrom_MACCS_75 | MACCS fingerprint |
| 546 | Chrom_MACCS_76 | MACCS fingerprint |
| 547 | Chrom_MACCS_77 | MACCS fingerprint |
| 548 | Chrom_MACCS_78 | MACCS fingerprint |
| 549 | Chrom_MACCS_79 | MACCS fingerprint |
| 550 | Chrom_MACCS_80 | MACCS fingerprint |
| 551 | Chrom_MACCS_81 | MACCS fingerprint |
| 552 | Chrom_MACCS_83 | MACCS fingerprint |
| 553 | Chrom_MACCS_85 | MACCS fingerprint |
| 554 | Chrom_MACCS_86 | MACCS fingerprint |
| 555 | Chrom_MACCS_87 | MACCS fingerprint |
| 556 | Chrom_MACCS_89 | MACCS fingerprint |
| 557 | Chrom_MACCS_92 | MACCS fingerprint |
| 558 | Chrom_MACCS_93 | MACCS fingerprint |
| 559 | Chrom_MACCS_94 | MACCS fingerprint |
| 560 | Chrom_MACCS_95 | MACCS fingerprint |
| 561 | Chrom_MACCS_96 | MACCS fingerprint |
| 562 | Chrom_MACCS_97 | MACCS fingerprint |
| 563 | Chrom_MACCS_98 | MACCS fingerprint |
| 564 | Chrom_MACCS_99 | MACCS fingerprint |
| 565 | Chrom_MACCS_100 | MACCS fingerprint |
| 566 | Chrom_MACCS_101 | MACCS fingerprint |
| 567 | Chrom_MACCS_105 | MACCS fingerprint |
| 568 | Chrom_MACCS_106 | MACCS fingerprint |
| 569 | Chrom_MACCS_108 | MACCS fingerprint |
| 570 | Chrom_MACCS_109 | MACCS fingerprint |
| 571 | Chrom_MACCS_111 | MACCS fingerprint |
| 572 | Chrom_MACCS_112 | MACCS fingerprint |
| 573 | Chrom_MACCS_113 | MACCS fingerprint |
| 574 | Chrom_MACCS_114 | MACCS fingerprint |
| 575 | Chrom_MACCS_115 | MACCS fingerprint |
| 576 | Chrom_MACCS_116 | MACCS fingerprint |
| 577 | Chrom_MACCS_117 | MACCS fingerprint |
| 578 | Chrom_MACCS_118 | MACCS fingerprint |
| 579 | Chrom_MACCS_119 | MACCS fingerprint |
| 580 | Chrom_MACCS_120 | MACCS fingerprint |
| 581 | Chrom_MACCS_121 | MACCS fingerprint |
| 582 | Chrom_MACCS_122 | MACCS fingerprint |
| 583 | Chrom_MACCS_123 | MACCS fingerprint |
| 584 | Chrom_MACCS_124 | MACCS fingerprint |
| 585 | Chrom_MACCS_127 | MACCS fingerprint |
| 586 | Chrom_MACCS_128 | MACCS fingerprint |
| 587 | Chrom_MACCS_129 | MACCS fingerprint |
| 588 | Chrom_MACCS_130 | MACCS fingerprint |
| 589 | Chrom_MACCS_133 | MACCS fingerprint |
| 590 | Chrom_MACCS_135 | MACCS fingerprint |
| 591 | Chrom_MACCS_136 | MACCS fingerprint |
| 592 | Chrom_MACCS_137 | MACCS fingerprint |
| 593 | Chrom_MACCS_138 | MACCS fingerprint |
| 594 | Chrom_MACCS_140 | MACCS fingerprint |
| 595 | Chrom_MACCS_141 | MACCS fingerprint |
| 596 | Chrom_MACCS_142 | MACCS fingerprint |
| 597 | Chrom_MACCS_143 | MACCS fingerprint |
| 598 | Chrom_MACCS_144 | MACCS fingerprint |
| 599 | Chrom_MACCS_145 | MACCS fingerprint |
| 600 | Chrom_MACCS_146 | MACCS fingerprint |
| 601 | Chrom_MACCS_148 | MACCS fingerprint |
| 602 | Chrom_MACCS_149 | MACCS fingerprint |
| 603 | Chrom_MACCS_150 | MACCS fingerprint |
| 604 | Chrom_MACCS_151 | MACCS fingerprint |
| 605 | Chrom_MACCS_152 | MACCS fingerprint |
| 606 | Chrom_MACCS_153 | MACCS fingerprint |
| 607 | Chrom_MACCS_154 | MACCS fingerprint |
| 608 | Chrom_MACCS_155 | MACCS fingerprint |
| 609 | Chrom_MACCS_156 | MACCS fingerprint |
| 610 | Chrom_MACCS_157 | MACCS fingerprint |
| 611 | Chrom_MACCS_158 | MACCS fingerprint |
| 612 | Chrom_MACCS_159 | MACCS fingerprint |
| 613 | Chrom_MACCS_160 | MACCS fingerprint |
| 614 | Chrom_MACCS_161 | MACCS fingerprint |
| 615 | Chrom_MACCS_164 | MACCS fingerprint |
| 616 | Solv_Morgan_13 | Morgan fingerprint |
| 617 | Solv_Morgan_33 | Morgan fingerprint |
| 618 | Solv_Morgan_80 | Morgan fingerprint |
| 619 | Solv_Morgan_114 | Morgan fingerprint |
| 620 | Solv_Morgan_295 | Morgan fingerprint |
| 621 | Solv_MACCS_112 | MACCS fingerprint |
| 622 | Solv_MACCS_150 | MACCS fingerprint |
| 623 | Solv_MACCS_153 | MACCS fingerprint |
| 624 | Cluster | Engineered / interaction descriptor |
| 625 | Chrom_KappaRatio | Engineered / interaction descriptor |
| 626 | Chrom_HbondPolarityScore | Engineered / interaction descriptor |
| 627 | Chrom_PolarityBalance | Engineered / interaction descriptor |
| 628 | Chrom_Sqrt_TPSA | Engineered / interaction descriptor |
| 629 | Solv_KappaRatio | Engineered / interaction descriptor |
| 630 | AbsEmiRatio | Engineered / interaction descriptor |
| 631 | AbsEmiRatio_sq | Engineered / interaction descriptor |
| 632 | AbsEmiRatio_cu | Engineered / interaction descriptor |
| 633 | StokesShift | Engineered / interaction descriptor |
| 634 | StokesShift_Ratio | Engineered / interaction descriptor |
| 635 | ChromSolv_LogP_Diff | Engineered / interaction descriptor |
| 636 | ChromSolv_TPSA_Diff | Engineered / interaction descriptor |
| 637 | Emission_Energy_eV | Engineered / interaction descriptor |
| 638 | Absorption_Energy_eV | Engineered / interaction descriptor |
| 639 | StokesShift_eV | Engineered / interaction descriptor |

# Validation of the model with TD-DFT methods

External comparison between ML predictions and TD-DFT Scheme C fluorescence lifetimes.  Comparison of ML-predicted fluorescence lifetimes with experimental values and TD-DFT Scheme C errors for non-overlapping chromophore–solvent pairs from the Wong et al. The table reports the ML prediction error together with the corresponding BMK, LC-BLYP*, and ωB97X* TD-DFT errors, keeping the notation from the article for the molecular structures.

Table S7. Independent external validation of the model with TD-DFT methods

| **Molecule** | **Class** | **Exp. lifetime (ns)** | **ML predicted (ns)** | **ML error (%)** | **BMK error (%)** | **LC-BLYP* error (%)** | **ωB97X* error (%)** |
| --- | --- | --- | --- | --- | --- | --- | --- |
| II | Fused aromatic ring | 11.50 | 10.550 | 8.26 | 34.35 | 9.74 | 2.44 |
| III | Fused aromatic ring | 5.50 | 6.782 | 23.31 | 29.46 | 9.46 | 12.00 |
| IV | Fused aromatic ring | 6.20 | 5.999 | 3.24 | 15.16 | 15.16 | 20.48 |
| V | Fused aromatic ring | 7.50 | 7.279 | 2.95 | 0.80 | 13.20 | 8.13 |
| VI | Acridone | 10.50 | 10.044 | 4.34 | 32.86 | 10.67 | 3.62 |
| VII | Acridone | 10.60 | 9.553 | 9.88 | 34.43 | 45.76 | 36.04 |
| VIII | Acridone | 10.70 | 6.840 | 36.08 | 5.33 | 16.73 | 3.36 |
| IX | Conjugated aromatic ring | 1.36 | 0.999 | 26.51 | 0.74 | 12.50 | 12.50 |
| XI | Conjugated aromatic ring | 0.64 | 2.249 | 251.39 | 31.25 | 45.31 | 40.63 |
| XIII | Bimane | 7.40 | 7.223 | 2.40 | 9.19 | 12.70 | 20.95 |
| XIV | Bimane | 9.80 | 6.021 | 38.56 | 15.10 | 7.25 | 18.98 |
| XV | Bimane | 17.00 | 8.714 | 48.74 | 26.59 | 40.06 | 13.53 |
| XVI | Bimane | 10.70 | 7.745 | 27.61 | 1.68 | 28.32 | 0.84 |
| XVII | Bimane | 12.30 | 7.420 | 39.67 | 14.47 | 1.38 | 19.92 |
| XVIII | Bimane | 9.00 | 9.624 | 6.94 | 24.22 | 13.22 | 24.33 |
| XIX | Coumarin | 5.20 | 5.866 | 12.80 | 1.35 | 10.96 | 10.00 |
| XX | Coumarin | 3.50 | 4.130 | 18.01 | 29.14 | 15.43 | 36.86 |
| XXII | Coumarin | 4.10 | 4.473 | 9.09 | 7.56 | 6.34 | 11.71 |
| XXIII | Coumarin | 5.40 | 5.905 | 9.35 | 32.22 | 24.63 | 37.04 |
| XXIV | Coumarin | 3.30 | 4.321 | 30.93 | 39.09 | 24.24 | 33.33 |

# External experimental validation data and references

The independent experimental validation set contained diverse chromophore families including perylene derivatives, cyanine/merocyanine dyes, BODIPY systems, coumarins, benzoxazole derivatives, flavin-like structures, and donor-acceptor oligomers. Table S7 reports the validation values used in the manuscript, and Table S8 links each tag to the corresponding experimental source DOI.

Table S8. Independent external experimental validation set. Difference = predicted lifetime - experimental lifetime.

| **Tag** | **Compound / structural description** | **Class** | **Solvent** | **Exp. lifetime (ns)** | **Pred. lifetime (ns)** | **Difference (ns)** | **Error (%)** |
| --- | --- | --- | --- | --- | --- | --- | --- |
| E01 | Butyl-substituted perylene | Perylene / PAH | Hexane | 69.100 | 29.715 | -39.385 | 57.00 |
| E02 | Triflylimino polycyclic xanthene-like dye | Polycyclic heteroaromatic dye | DMSO | 6.600 | 4.593 | -2.007 | 30.41 |
| E03 | Pyridyl-substituted heteroaromatic dye | N-heteroaromatic donor-acceptor dye | DMF | 1.910 | 2.544 | 0.634 | 33.19 |
| E04 | Bis(julolidine) cyclohexanone merocyanine | Cyanine / merocyanine dye | DMF | 0.750 | 0.674 | -0.076 | 10.13 |
| E05 | Tricyano phenylene-ethynylene oligomer | Cyano-substituted conjugated oligomer | Chloroform | 1.360 | 1.261 | -0.099 | 7.28 |
| E06 | Triisopropylsilyl-ethynyl azaacene derivative | Extended azaacene / NIR dye | Dichloromethane | 18.300 | 7.397 | -10.903 | 59.58 |
| E07 | Boc-amino-acid benzoxazole-anthracene derivative | Benzoxazole / amino-acid fluorophore | Methylcyclohexane | 2.700 | 2.534 | -0.166 | 6.15 |
| E08 | Boc-amino-acid benzoxazole-carbazole derivative | Benzoxazole / carbazole fluorophore | 2-Methyltetrahydrofuran | 1.830 | 1.864 | 0.034 | 1.86 |
| E09 | Boc-amino-acid benzoxazole-nitrile derivative | Benzoxazole fluorophore | Methylcyclohexane | 1.280 | 1.161 | -0.119 | 9.30 |
| E10 | N-ribosyl flavin derivative | Flavin / isoalloxazine dye | Acetonitrile | 3.610 | 3.941 | 0.331 | 9.17 |
| E11 | Bis-styrylpyridinium naphthalene dye | Hemicyanine / styrylpyridinium dye | Toluene | 2.740 | 3.360 | 0.620 | 22.63 |
| E12 | Alkoxy oligothiophene-benzothiadiazole oligomer | Donor-acceptor oligothiophene | Toluene | 4.500 | 5.557 | 1.057 | 23.49 |
| E13 | Vinyl-methylenedioxycoumarin | Coumarin / furocoumarin-type dye | Acetonitrile | 3.680 | 5.519 | 1.839 | 49.97 |
| E14 | Methyl perylene carboxylate | Perylene / PAH ester | Acetonitrile | 9.360 | 9.577 | 0.217 | 2.32 |
| E15 | Tetramethyl-BODIPY derivative | BODIPY dye | Hexane | 5.850 | 6.140 | 0.290 | 4.96 |
| E16 | Carbazole-iodo-BODIPY conjugate | Heavy-atom BODIPY dye | Chloroform | 3.930 | 3.878 | -0.052 | 1.32 |
| E17 | Thiophene-rich polymethine dye | Oligothiophene / polymethine dye | 1,4-Dioxane | 0.390 | 0.286 | -0.104 | 26.67 |
| E18 | 5-Aminoisatin | Isatin / indole-dione dye | THF | 12.400 | 15.227 | 2.827 | 22.80 |

Table S9. Experimental source references for the independent external validation set.

| **Tag** | **Compound / structural description** | **Solvent** | **Experimental source DOI** |
| --- | --- | --- | --- |
| E01 | Butyl-substituted perylene | Hexane | https://doi.org/10.1021/jo400128c |
| E02 | Triflylimino polycyclic xanthene-like dye | DMSO | https://doi.org/10.1021/acs.orglett.5b02042 |
| E03 | Pyridyl-substituted heteroaromatic dye | DMF | https://doi.org/10.1021/jp512583m |
| E04 | Bis(julolidine) cyclohexanone merocyanine | DMF | https://doi.org/10.1016/j.molstruc.2016.02.009 |
| E05 | Tricyano phenylene-ethynylene oligomer | Chloroform | https://doi.org/10.1021/ol0528991 |
| E06 | Triisopropylsilyl-ethynyl azaacene derivative | Dichloromethane | https://doi.org/10.1021/jo502564w |
| E07 | Boc-amino-acid benzoxazole-anthracene derivative | Methylcyclohexane | https://doi.org/10.1016/j.jphotochem.2004.08.015 |
| E08 | Boc-amino-acid benzoxazole-carbazole derivative | 2-Methyltetrahydrofuran | https://doi.org/10.1039/c2pp25114k |
| E09 | Boc-amino-acid benzoxazole-nitrile derivative | Methylcyclohexane | https://doi.org/10.1016/j.jphotochem.2005.04.034 |
| E10 | N-ribosyl flavin derivative | Acetonitrile | https://doi.org/10.1016/1010-6030(94)03924-J |
| E11 | Bis-styrylpyridinium naphthalene dye | Toluene | https://doi.org/10.1016/S0040-4039(00)01039-X |
| E12 | Alkoxy oligothiophene-benzothiadiazole oligomer | Toluene | https://doi.org/10.1021/acs.jpcc.6b07290 |
| E13 | Vinyl-methylenedioxycoumarin | Acetonitrile | https://doi.org/10.1021/ol201983u |
| E14 | Methyl perylene carboxylate | Acetonitrile | https://doi.org/10.1016/j.tet.2012.05.072 |
| E15 | Tetramethyl-BODIPY derivative | Hexane | https://doi.org/10.1007/s10895-019-02349-5 |
| E16 | Carbazole-iodo-BODIPY conjugate | Chloroform | https://doi.org/10.1021/jo401379g |
| E17 | Thiophene-rich polymethine dye | 1,4-Dioxane | https://doi.org/10.1016/j.jlumin.2017.03.042 |
| E18 | 5-Aminoisatin | THF | https://doi.org/10.1021/jp9526509 |
